# Artificial Intelligence and Acute Appendicitis: A Systematic Review of Diagnostic and Prognostic Models

**DOI:** 10.1186/s13017-023-00527-2

**Published:** 2023-12-19

**Authors:** Mahbod Issaiy, Diana Zarei, Amene Saghazadeh

**Affiliations:** 1 School of Medicine, Tehran University of Medical Sciences (TUMS), Tehran, Iran; 2 Systematic Review and Meta-Analysis Expert Group (SRMEG), Universal Scientific Education and Research Network (USERN), Tehran, Iran; 3 School of Medicine, Iran University of Medical Sciences, Tehran, Iran; 4 Advanced Diagnostic and Interventional Radiology Research Center (ADIR), Tehran University of Medical Science, Tehran, Iran; 5 Research Center for Immunodeficiencies, Children’s Medical Center, Tehran University of Medical Sciences, Tehran, Iran

**Keywords:** Acute appendicitis, AI, Deep learning, Machine learning, Systematic review

## Abstract

**Background:**

To assess the efficacy of artificial intelligence (AI) models in diagnosing and prognosticating acute appendicitis (AA) in adult patients compared to traditional methods. AA is a common cause of emergency department visits and abdominal surgeries. It is typically diagnosed through clinical assessments, laboratory tests, and imaging studies. However, traditional diagnostic methods can be time-consuming and inaccurate. Machine learning models have shown promise in improving diagnostic accuracy and predicting outcomes.

**Main body:**

A systematic review following the PRISMA guidelines was conducted, searching PubMed, Embase, Scopus, and Web of Science databases. Studies were evaluated for risk of bias using the Prediction Model Risk of Bias Assessment Tool. Data points extracted included model type, input features, validation strategies, and key performance metrics.

**Results:**

In total, 29 studies were analyzed, out of which 21 focused on diagnosis, seven on prognosis, and one on both. Artificial neural networks (ANNs) were the most commonly employed algorithm for diagnosis. Both ANN and logistic regression were also widely used for categorizing types of AA. ANNs showed high performance in most cases, with accuracy rates often exceeding 80% and AUC values peaking at 0.985. The models also demonstrated promising results in predicting postoperative outcomes such as sepsis risk and ICU admission. Risk of bias was identified in a majority of studies, with selection bias and lack of internal validation being the most common issues.

**Conclusion:**

AI algorithms demonstrate significant promise in diagnosing and prognosticating AA, often surpassing traditional methods and clinical scores such as the Alvarado scoring system in terms of speed and accuracy.

**Supplementary Information:**

The online version contains supplementary material available at 10.1186/s13017-023-00527-2.

## Introduction

Acute abdominal pain constitutes 7–10% of all emergency department visits. Acute appendicitis (AA) is a prevalent etiology of lower abdominal pain, prompting individuals to seek emergency care, and is the predominant diagnosis in young patients hospitalized for acute abdominal conditions [1]. Although the majority of cases manifest acutely within a 24-h frame, some can evolve into chronic conditions [2]. AA can be further stratified into distinct categories, namely simple, perforated, and gangrenous forms [3]. Primarily affecting individuals between the ages of 5 and 45, appendicitis has an incidence rate of about 233 per 100,000 people. It is slightly more prevalent in males, with a lifetime risk of 8.6% compared to 6.7% in females [4].

Traditionally, appendicitis diagnosis has relied on a combination of clinical evaluation, laboratory tests, and imaging studies, including ultrasound and computed tomography (CT) scans [5]. However, these methods are fraught with limitations, such as diagnostic inaccuracies and time-consuming procedures, which could result in severe complications like appendix perforation and sepsis [6].

To overcome these challenges, advancements in artificial intelligence (AI) have begun to augment conventional diagnostic frameworks. AI refers to machine capabilities that simulate human cognitive processes to perform tasks autonomously [7].

The terms AI, machine learning (ML), and deep learning (DL) represent a nested hierarchy of intelligent systems, where DL is a specialized subtype of ML, which itself falls under the broader category of AI [8]. In some studies, ML techniques like support vector machines (SVM) and random forests (RF) have been utilized for classification tasks. These techniques enhance diagnostic precision by learning from data and experience [9, 10]. DL involves using multilayer (“deep”) neural networks for data-driven computation and processing [11]. DL architectures like convolutional neural networks (CNNs) have demonstrated superior performance in analyzing intricate patterns in imaging data, occasionally surpassing human-level expertise [12]. Natural language processing (NLP) algorithms are another AI avenue that is applied to extract relevant clinical information from electronic health records, aiding in diagnostic and prognostic evaluations [13]. Reinforcement learning models have also been explored for their potential to optimize treatment strategies, such as deciding between surgical intervention and conservative management, by simulating various clinical scenarios [14]. Furthermore, ensemble techniques that amalgamate various AI models have emerged to offer more reliable and robust diagnostic solutions [15].

In light of these innovations, there has been an increasing number of studies focusing on the potential of AI in the diagnosis and management of appendicitis, exploring a multitude of input variables and ML approaches [12]. Despite these promising advances, the acute and potentially life-threatening nature of appendicitis underscores the necessity for highly reliable and efficacious AI algorithms [6, 16].

In this systematic review, we investigate how AI contributes to diagnosing AA and predicting its outcomes. We aim to assess the effectiveness of different AI models and compare them to traditional methods in diagnosing AA, classifying its types, and forecasting the outcomes after surgery.

## Methods

### Study Design

This study is a systematic review aimed at evaluating the applications of AI in the diagnosis and prognosis of appendicitis in adult patients. The review follows the Preferred Reporting Items for Systematic Reviews and Meta-Analyses (PRISMA) guidelines [17] (Additional file 1). Furthermore, the study protocol has been formally registered in the International Prospective Register of Systematic Reviews, with the identification number CRD42023444627.

### Search Strategy

We conducted a thorough systematic search in PubMed, Embase, Scopus, and Web of Science databases using a set of keywords pertinent to “artificial intelligence” and “appendicitis.” The search was designed to include articles published up to August 2023. A comprehensive description of the search strategy for each database can be found in Additional file 2.

### Study Selection and Eligibility Criteria

Two independent reviewers (M.I. and D.Z.) initially screened the search results based on titles and abstracts. After this initial screening, full-text articles were carefully examined for their relevance. Inclusion criteria encompassed original research articles that were peer-reviewed and focused on the application of any AI-based model in diagnosing or prognosticating appendicitis. Exclusion criteria included studies specifically dealing with pediatric appendicitis, those relying on pre-existing external databases, those with insufficient data, or those that trained their models only using radiology reports or clinicians’ notes. Articles such as case reports, reviews, conference proceedings, and editorials were also excluded.

### Data Extraction

Data extraction was carried out independently by two reviewers (M.I. and D.Z.). In cases where discrepancies arose, a third reviewer was consulted to reach a consensus (A.S.). The data points extracted included study design, primary objectives, sample size, data sources, reference standards, input features used for model training, techniques to address data imbalance, types of algorithms employed, preprocessing measures, model training and validation strategies, comparator models, key performance metrics like AUC, sensitivity, specificity, and accuracy, key findings, and limitations.

### Risk of Bias Assessment

The studies included in the research were evaluated using the Prediction Model Risk of Bias Assessment Tool (PROBAST), a tool designed to assess the risk of bias across four distinct domains while also evaluating the applicability of diagnostic and prognostic models within the research context [18]. The quality assessment was conducted independently by two authors (M.I. and D.Z.), and any discrepancies were resolved by a third author (A.S.). Based on the established criteria of the PROBAST tool, the studies were then classified into one of three risk categories: low, unclear, or high. In this context, a study was considered to have a high risk of bias if it received a high-risk classification in any one of the four domains assessed by PROBAST.

### Data Synthesis and Analysis

Studies were categorized based on their primary focus: diagnosis or prognosis. Whenever possible, a meta-analysis will be conducted; however, if heterogeneity or diverse input variables preclude this, findings will be presented descriptively and categorically.

## Results

### Study Selection

Our initial search across multiple databases led to the identification of 628 articles. An additional four articles were included from auxiliary sources. Following the removal of duplicates, 382 articles remained for screening. After a comprehensive review of titles and abstracts, 84 articles were deemed eligible based on our predetermined criteria. Following an in-depth examination of the full texts, 29 articles ultimately met the inclusion standards. The flowchart in Additional file 3: Figure S1 delineates each step of the article selection process.

### Characteristics of the Studies

Among the 29 articles that met the inclusion criteria, 21 articles (72%) focused on the diagnosis of AA, seven (24%) on its prognosis, and one article addressed both diagnosis and prognosis [19]. Over half of the studies (51%) adopted a cross-sectional research design. Furthermore, a substantial portion of the included studies, 16 of them (55%), were conducted within the past 5 years (2019 and later). Remarkably, all prognosis-related studies were carried out within the last 3 years (2021 and later). A comprehensive overview can be found in Additional file 4: Figure S2.

### Risk of Bias Assessment

**Fig. 1:**
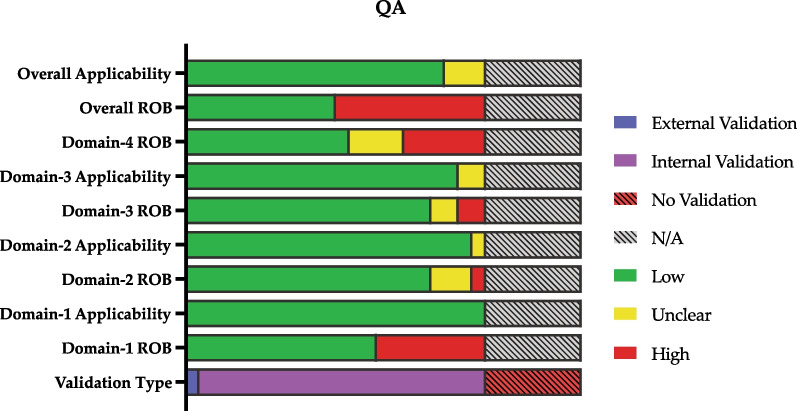
Quality assessment of included studies

Utilizing the PROBAST, our assessment revealed that among the reviewed studies, 11 exhibited low risk of bias [3, 12, 19–27], while 18 exhibited high risks of bias [28–45]. The primary factor contributing to a high risk of bias was selection bias [30, 32, 33, 36, 39, 42, 44, 45], identified in eight studies. Furthermore, seven studies lacked internal validation and thus were excluded from further quality assessment [34, 35, 37, 38, 40, 41, 43]. Additionally, six studies had issues related to their analyses [29–32, 42, 44]. In two studies, a high risk of bias was associated with the outcome or its determination [28, 32]. One study had a high risk of bias introduced by their predictors or assessment [32]. The comprehensive evaluation of each domain’s quality across the studies is illustrated in Fig. 1. For a detailed breakdown of the quality assessment and PROBAST domains, please refer to Additional file 5: Table S1.

### Artificial Intelligence Algorithms

A total of 24 distinct artificial intelligence algorithms were applied across the selected articles. These algorithms fell into six main categories: (1) Statistical classifiers, encompassing Logistic Regression (LR) and Naïve Bayes (NB); (2) ML classifiers such as SVM, Decision Trees (DT), and K-Nearest Neighbors (KNN); (3) Ensemble ML techniques, which include RFs, Pre-clustering Ensemble Learning (PEL), and variants of Boosted DT like Gradient Boosting (GB), Extreme Gradient Boosting (XGB), and CatBoost; (4) ML Neural Networks, including Artificial Neural Networks (ANN) and other specialized forms like Multilayer Perceptron (MLP), Backpropagation Neural Network (BPNN), Radial Basis Function Network (RBFN), Adaptive Resonance Theory (ART), Self-Organizing Maps (SOMs), Learning Vector Quantization (LVQ), Multilayer Neural Network (MLNN), Probabilistic Neural Network (PNN), Extreme Learning Machines (ELM), and Kernel ELM (KELM); (5) DL techniques, particularly CNNs; and (6) other miscellaneous algorithms including fuzzy rule-based and neuro-fuzzy approaches.

Over half of the studies (51%) utilized a singular algorithmic model, while the remainder employed multiple models, varying from two [26, 40, 43, 44] to six [25] in number. Comprehensive data on the types of models employed are outlined in Tables 1 and 3.

**Table 1:** Characteristics of studies in artificial intelligence applications for appendicitis diagnosis

Study, year	Objective(s)	Algorithm(s) applied	Sample size (training: validation)	Age, mean ± SD (year)	Sex (male/female)
Park et al. [1], 2023 (South Korea)	Develop a CNN model for automated classification of AA	CNN	715 cases in total (4078 CT images) 246 cases had AA (1959 CT images) Total cases (568:147) AA cases (199:47)	Total patients: 44.3 ± 18.4 AA: 41.9 ± 19.2	Total patients: 368/347 AA: 130/116
Akbulut et al. [2], 2023 (Turkey)	Develop an ML model for the classification of AA and predicting perforated and non-perforated AA	CatBoost	1797 (80%/20%) (AA: 1465, NA: 332, AA non-perforated 1161, AA perforated: 304)	Male (median: 33; IQR: 23). female (median: 34; IQR: 26). AA (median: 33.1; IQR: 25), NA (median: 33; IQR: 24)	993/804
Ghareeb et al. [3], 2021 (Egypt)	Determine how well an AI-based model performs in diagnosing AA compared to using the Alvarado score alone or in combination with ultrasound criteria	Subspace KNN, KNN, LR, DT, SVM, NB	319 (224: 95)	30.5 ± 12.5	47.6%/52.4%
Rajpurkar et al. [4], 2020 (USA)	Develop a 3D DL model called “AppendiXNet” for appendicitis detection using a small training dataset of under 500 CT examinations	CNN	Training: 438 examinations (255 appendicitis and 183 NA from 435 patients), Development: 106 examinations (53 appendicitis and 53 NA from 105 patients) Test: 102 examinations (51 appendicitis and 51 NA from 102 patients)	Training: 38.2 ± 15.6 development: 39.2 ± 17.3 Test: 38.4 ± 15.7	Training: 182/253 Developmental: 41/64 Test: 38/64
Park et al. [5], 2020 (USA)	Examine the viability of a CNN-based diagnostic algorithm for AA using abdominopelvic CT scans	CNN	Training and internal validation: 667 (215 AA, 452 NA), External validation: Institution 1: 60 patients (26 AA, 34 NA), Institution 2: 40 patients (20 AA, 20 NA)	Training and internal validation: 45.6 ± 22.2 External validation: Institution1 (45.9 ± 18.9), Institution2 (43.9 ± 20.8)	Training and internal validation: 331/336, External validation: Institution 1 (25/35), Institution 2 (24/16)
Zhao et al. [6], 2020 (China)	Provide potential urinary markers and an efficient model for AA diagnosis	Naïve Bayes, SVM, and RF	134: 73/61 (48 patients had AA and 86 patients had other acute abdomens) AA (32 training: 16 validation) NA (41 training: 45 validation) 495 healthy controls	AA (training: 38:5 ± 17:9, validation: 34:2 ± 15:2), NA (training: 55:1 ± 18:2, validation: 56:6 ± 17:2)	AA (training: 14/18, validation: 9/7), NA (training: 13/28, validation: 14/17)
Ramirez garcialunaa et al. [7], 2020 (Mexico)	Evaluate skin IRT imaging as a diagnostic adjunct for AA in adults	RF	122: 98/24 (71 patients: 51 had AA, 20 patients had other diagnoses. 51 healthy controls)	AA: 29.1 ± 14.1, other patients: 30.0 ± 11.8, Healthy controls: 21.0 ± 3.3	AA: 25/26, Other patients: 5/15, Healthy control: 31/20
Kang et al. [8], 2019 (South Korea)	Assess the effectiveness of a clinical approach using DT for diagnosis and compare its diagnostic performance with existing scoring systems	DT	244 (80 patients with AA, 164 patients with other diagnoses)	AA: 35 (23–42) NA: 31 (23–41)	AA: 31/49 NA: 54/109
Gudelis et al. [9], 2019 (Spain)	Develop a diagnostic model for RIF pain. Specifically, the study focuses on creating a diagnosis model based on classification trees using the CHAID method and an ANN	ANN (MLP + BP), DT	252 (93 patients had AA)	Total: 33.3 ± 16 AA: 37 ± 17	Total: 52.8%/47.2% AA: 74.2%/25.6%
Shahmoradi et al. [10], 2018 (Iran)	Construct a model to predict AA based on pathology reports	Radial Basis Function Network (RBFN), MLP, and LR	181 cases (133 patients had AA)	Average: 28	126/55
Jamshidnezhad et al. [11], 2017 (Iran)	Develop a diagnostic model using minimal clinical factors within the initial hours of abdominal pain	Fuzzy-rule-based system (using Honey Bee Reproduction Cycle (HRBC))	70%/30%	NR	NR
Park et al. [12], 2015 (South Korea)	Propose an appendicitis diagnosis system using ANN	RBFNN, MLNN, PNN	801 cases I. NA (N = 596) II. Appendicitis (N = 205, No AA (N = 143), AA (n = 62))	Total: 30.27 ± 18.58, NA: 29.68 ± 13.63, No AA: 30.55 ± 13.99 AA: 31.53 ± 16.32	NA: 290/306, No AA: 53/90, AA: 32/30
Safavi et al. [13], 2015 (Iran)	Compare the ANN models and conventional laboratory tests in the diagnosis of appendicitis	ANN (MLP)	100 (83 AA, 17 NA): training: 60, validation: 15, testing: 25	28/01 ± 12/68	71/29
Lee et al. [14], 2013 (Southern Taiwan)	Evaluate the prediction effectiveness of the PEL technique, addressing imbalanced sample learning issues, to support accurate diagnosis of AA	PEL	574 (464 positive appendicitis, 110 negative appendicitis)	36.18 (3–87) Positive appendicitis: 36.97 Negative appendicitis: 32.5	323/251
Yoldaş et al. [15], 2012 (Turkey)	Create a diagnostic model with ANNs and assess its effectiveness in diagnosing AA	ANN	156 (132 appendicitis, 24 NA)	Total: 29.9 ± 10.8, Appendicitis: 29.3 ± 10.6, NA: 33.2 ± 11.9	Total: 79/77, Appendicitis: 72/60, NA: 7/17
Sun et al. [16], 2012 (South Korea)	Build a hybrid decision support model to accurately diagnose suspected AA and identify useful decision rules by combining statistical analysis and DT algorithms	DT	326 cases (90%:10%): 152 AA, 174 NA	AA: 36.57 ± 21.31 NA: 43.05 ± 20.86	AA: 77/75 NA: 66/108
Hsieh et al. [17], 2011 (Taiwan)	Evaluate the performance of RF, SVM, and ANN in diagnosing AA	RF, SVM, ANN, LR	180 (135:45), 115 patients had appendicitis	39.4 (16–85)	85/95
Ting et al. [18], 2010 (Taiwan)	Modify the ASS with decision tree technology and construct a convenient and accurate decision support model for AA diagnosis and timing of laparotomy	DT	532 (340 patients had AA, 80 had perforated appendicitis, 112 NA)	AA: 31.9 NA: 29.9 Ruptured appendicitis: 37.1	Total: 327/205
Prabhudesai et al. [19], 2008 (UK)	Assess the use of ANN for diagnosing appendicitis in patients with acute right iliac fossa pain	MLP type ANN	60 patients with suspected appendicitis: 24 had appendicitis, 36 had other diagnosis	25.4	27/33
Sakai et al. [20], 2007 (Japan)	Compare the diagnostic accuracy levels of ANN models and LR models for diagnosing AA	ANN and LR	169 (86 cases had AA, 83 cases NA)	AA: 24.4 ± 20.3, NA: 27.5 ± 17.4	77/92 AA: 42/44, NA: 35/48
Pesonen et al. [21], 1996 (Finland)	Compare four ANN algorithms for diagnosing AA	Four different types of NN: two unsupervised learning and feedback networks including binary ART1 and Kohonen SOM. LVQ (supervised learning and feedback network), and BP (supervised learning and feedforward network)	911 (454:457)	NR	47.7%/52.3%
Forsstrom et al. [22], 1995 (Finland)	Create the DIAGAID software package to establish a SMARTTP link connecting patient databases and clinicians	Neuro-fuzzy systems (DiagaiD), BPNN, LR	186 (120:66), 145 patients had AA, and 41 did not have)	NR	NR

*CNN* convolutional neural network, *AA* acute appendicitis, *RF* random forest, *NLP* natural language processing, *NA* no appendicitis, *NR* not reported, *No AA* no acute appendicitis, *DL* deep learning, *DT* decision tree, *SVM* support vector machine, *ANN* artificial neural network, *US* ultrasonography, *MLP* multilayer perceptron, *MLNN* multilayer neural network, *PNN* probabilistic neural network, *RBF* radial basis function, *SOM* self-organizing map, *BP* backpropagation, *LVQ* learning vector quantization, *LR* logistic regression, *PEL* pre-clustering based ensemble learning, *ART* adaptive resonance theory, *CHAID* chi-square automatic interaction detection, *RIF* right iliac fossa

The ANN model and its variants were the most commonly employed, being featured in 13 studies [21, 24–27, 32–34, 37, 38, 40, 42, 45]. LR followed in frequency, appearing in nine studies [3, 24–26, 28, 30, 32, 42, 43]. DT [25, 30, 35, 36, 40, 41, 43] and SVM [23, 24, 28, 30, 37, 45] were each utilized in seven studies, while RF [23, 24, 29, 31, 45] was implemented in six studies.

**Fig. 2:**
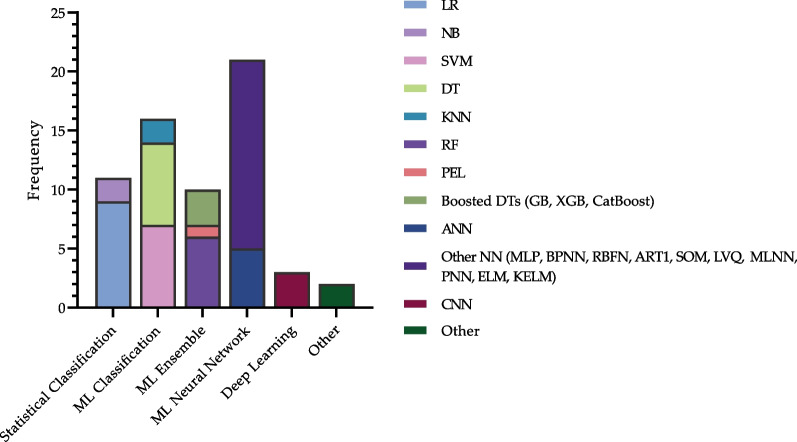
Algorithms utilized in studies

**Fig. 3:**
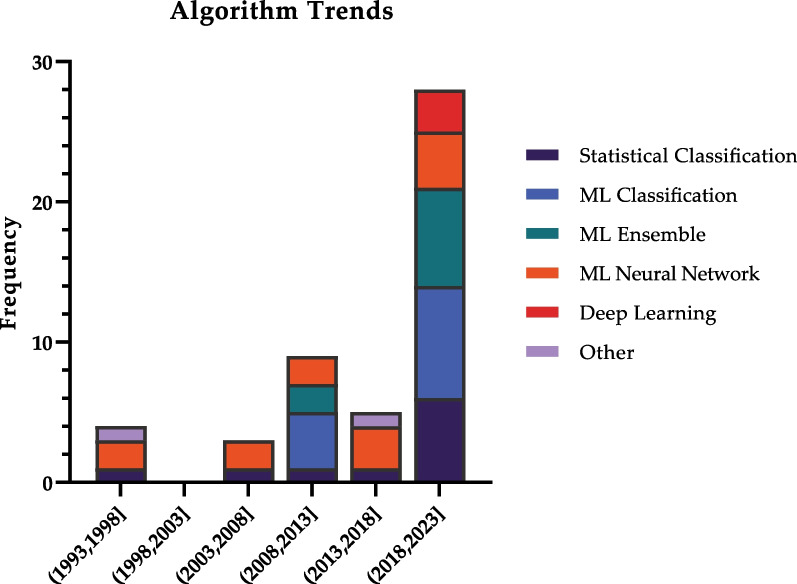
Trends of different algorithms

The distribution of algorithms and their subtypes is outlined in Fig. 2, and temporal trends are depicted in Fig. 3.

### Diagnosis of Appendicitis

A total of 22 studies applied algorithms for the diagnosis of AA. The detailed information on the included studies is summarized in Tables 1 and 2.

**Table 2:** Details of artificial intelligence methods applied and outcomes in studies for appendicitis diagnosis

Study, year	Input features	Training/validation strategy	Performance	Comparative algorithms and scoring metrics	Key findings	Limitations
Park et al. [1], 2023 (South Korea)	CT slices	DL model trained using fivefold cross-validation and separate test dataset Each fold had 60–70% training samples, 15–20% validation samples for parameter tuning, and 15–25% test samples for final evaluation	Single-Slice method: Sensitivity: 85.6%, Specificity: 96%, PPV: 85.4%, Accuracy: 86.1%, AUC: 0.937 RGB method: Sensitivity: 87.8%, Specificity: 88%, PPV: 87.1%, Accuracy: 87.9%, AUC: 0.951	NR	CNN performed better with serial slices and the RGB method than with a single-slice method	1. Retrospective study 2. Limited acute diverticulitis CT images and data augmentation used for balance 3. Excluded complicated diverticulitis cases 4. No tool was developed for condition localization in CT images 5. CNN performance was not evaluated with coronal reformatted CT images
Akbulut et al. [2], 2023 (Turkey)	TBil, WBC, Neutrophil, WLR, NLR, CRP, and WNR values and lower PNR, PDW, and MCV	The persistence method was repeated 50 times with different seeds for model robustness. CatBoost model predicted AA, with optimized hyperparameters using grid search with tenfold cross-validation and 5 replicates	CatBoost: Sensitivity 84.2%, Specificity 93.2%, AUC 0.947, Accuracy 88.2%, F1-score 88.7%	NR	The CatBoost ML model demonstrated high accuracy in distinguishing between AA and NA patients, achieving an 88.2% accuracy rate	1. The study is retrospective and lacks comprehensive clinical data 2. Radiological data are missing for approximately 11% of the patient sample 3. Conducted at a single institution
Ghareeb et al. [3], 2021 (Egypt)	Age, gender, marital status, obesity, diabetes mellitus, hypertension, hepatitis B virus infection, hepatitis C virus infection, autoimmune diseases, pain history of similar, duration of pain, site of pain, nausea, vomiting, anorexia, body temperature, CBC, Hg, ultrasound findings	It assessed various learning algorithms and selected the best-performing model based on accuracy and AUC. Principal Component Analysis (PCA) was used for precise feature selection without excluding any variables. An optimization process reduced prediction errors, and external validation was done with a separate dataset. Variable importance was ranked, and Ensemble Bag optimization with 30 iterations minimized diagnostic classification errors to 0.129	The best model performance (Subspace KNN model): Sensitivity: 100%, Specificity: 80%, PPV: 97.9%, NPV: 96.7%, Accuracy: 91.1, AUC: 0.82 Other models accuracies: DT: 84.4%, LR: 87.5%, NB: 88.8%, SVM: 89.3%, KNN: 89.3%	Alvarado score: Sensitivity: 68.2%, Specificity: 80%, PPV: 96.7%, NPV: 22.9%, Accuracy: 69.5% US alone: Sensitivity: 50.8%, Specificity: 73.5%, PPV: 94.7%, NPV: 16.9%, Accuracy: 58.6% Combined US and Alvarado: Sensitivity: 69.6%, Specificity: 100%, PPV: 100%, NPV: 28%, Accuracy: 72.8%	1. The diagnostic accuracy of the AI model outperforms both the Alvarado score alone and the Alvarado score combined with US criteria 2. The AI model excels in diagnostic accuracy, except for specificity, which is higher when combined with specific criteria	1. Single-center study 2. Small number of patients 3. Exclusion of patients with colon cancer 4. Limited real-world 5. applicability of the AI model 6. Inclusion of patients with pathologies other than appendicitis may affect results
Rajpurkar et al. [4], 2020, (USA)	CT scan	Created development and test sets using stratified random sampling with a balance of about 50% appendicitis examinations and 50% non-appendicitis examinations	Pretrained on video images: Sensitivity: 78.4%, Specificity: 66.7%, Accuracy: 72.5%, AUC: 0.810 Not pretrained on video images: Sensitivity: 78.4%, Specificity: 35.3%, Accuracy: 56.9%, AUC: 0.724	NR	1. Small training dataset used; video pretraining compensates for dataset size 2. Model technique applicable for future medical image DL studies	1. Small training dataset, no investigation into video pretraining’s impact with data size 2. Pretraining model effect explored using Kinetics dataset 3. Single-center study 4. The model does not differentiate between CA and UCA
Park et al. [5], 2020 (USA)	CT scan	Used eightfold cross-validation. The dataset is split into 8 parts, 7 for training, and 1 for testing. Hyperparameters set based on initial training, used for all 8 models. External validation with CT data from two institutions on 8 trained CNN models. The deep CNN used in the algorithm was built with six convolutional layers, three max-pooling layers, and two fully connected layers	Training and internal validation: Sensitivity: 90.2%, Specificity: 92%, Accuracy: 91.5% External validation, institution 1 (Sensitivity: 88.5%, Specificity: 91.2%, Accuracy: 90%), institution 2 (Sensitivity: 95%, Specificity: 100%, Accuracy: 97.5%)	NR	Feasibility of CNN-based diagnosis algorithm for diagnosing acute appendicitis using CT data	1. Excluded patients with tumors in the appendix who had surgical removal 2. Trained and tested network using manually extracted 4 cm^3^ appendix region data
Zhao et al. [6], 2020 (China)	More than 800 proteins in each urine sample	Detected outliers in the discovery dataset (AA outliers and CON outliers) against a normal urine database (495 samples) to identify markers indicating changes under pathological conditions	RF model: Sensitivity: 81.2%, Specificity: 84.4%, Accuracy: 83.6% SVM model: Sensitivity: 25%, Specificity: 97.8%, Accuracy: 78.7%, NB model: Sensitivity: 68.8%, Specificity: 71.1%, Accuracy: 70%	NR	1. The urinary proteomic system finds markers for AA vs. other acute abdomens 2. The RF model has high specificity in AA diagnosis without clinical signs 3. Noninvasive urinary markers have potential for clinical use	1. No validation with a larger sample size 2. No absolute quantification for feature proteins 3. No exploration of combining urinary markers with metabolites
Ramirez garcialunaa et al. [7], 2020 (Mexico)	Abdominal skin IRT images	Training and validation cohorts had balanced distributions of patients in three categories (“healthy,” “appendicitis,” and “no appendicitis”) with nine relevant predictors. The final model was built by considering the accuracy-complexity trade-off	RF model: Accuracy: 76.9%, Sensitivity: 91.3%, Specificity: 56.3%, PPV: 75%, NPV: 81.8%	NR	1. IRT may complement diagnostic workup for appendicitis 2. IRT is a timesaving, low-cost, noninvasive imaging modality 3. IRT has the potential to improve the clinical decision-making process	1. Group sizes unequal, non-appendicitis smaller 2. Minimal clinical/laboratory differences between groups 3. No IRT vs. CT scan comparison, gold standard
Kang et al. [8], 2019 (South Korea)	Rebound tenderness severity, migration, urinalysis, symptom duration, leukocytosis, neutrophil count, and CRP levels	The DT comprises 11 final nodes. The severity of rebound tenderness was selected in the parent node	DT model: AUC: 0.85, [95% CI; 0.799–0.893]	Alvarado score: AUC: 0.695 AAS score: AUC: 0.749 Eskelinen score: AUC: 0.715	1. New clinical approach using DT aids AA diagnosis in adults with equivocal CT findings 2. Helps decide the disposition of patients with equivocal results	1. retrospective design, 2. A small patient population in some DT nodes 3. CT findings interpreted by only one radiologist
Gudelis et al. [9], 2019 (Spain)	Blumberg sign, pain migration, increased pain, increased pain with movement, pain when coughing, anorexia, temperature, number of leukocytes, hours of evolution, and CRP levels	The training and validation method involved implementing the ANN model using the Alyuda1 (Neurointelligence) program, which utilizes MLP methodology with BP. In this process, all candidate variables were included in the “full model” type. The models had automatic variable selection capabilities based on significance or hierarchy. Internal validation was conducted using cross-validation with 10 partitions	ANN model: for all diagnoses: PCC: 75%, for AA diagnosis: PCC: 93.5%, AUC: 0.95	CHAID: for all diagnoses: PCC: 74.2%, for AA diagnosis: PCC: 81.7%, AUC: 0.93	1. Professionals treating RIF pain can benefit from interpretable models 2. The CHAID model offers a classification with more than two possibilities (AA vs. non-AA) 3. Validation in a larger series is necessary to confirm the model’s performance	1. Assignment of groups not validated by literature 2. Small sample size, particularly in the NIRIF and IRIF diagnostic groups 3. Limited capacity of models that only compare AA versus other conditions in real patient management
Shahmoradi et al. [10], 2018 (Iran)	Demographic, symptoms, clinical signs, laboratory findings	Used an MLP network with two hidden layers (7 and 5 neurons) and specific activation functions. For RBFN, specific activation functions and rescaling methods were applied	MLP model: Sensitivity: 80%, Specificity: 97.5%, PPV: 92.3%, NPV: 93%, Accuracy: 92.9%, AUC: 0.832 RBFN model: Sensitivity: 28%, Specificity: 87.8%, PPV: 64.2%, NPV: 81.8%, Accuracy: 77.6%, AUC: NR LR model: Sensitivity: 58.3%, Specificity: 93.2%, PPV: 75.7%, NPV: 86%, Accuracy: 83.9%, AUC: 0.808	NR	1. MLP model outperforms LR in sensitivity, specificity, and accuracy 2. Essential predictors: leukocytosis, sex, tenderness, right iliac fossa pain	1. Small sample size: 2. Lack of imaging techniques 3. Risk of misdiagnosis 4. The study Limited variables
Jamshidnezhad et al. [11], 2017 (Iran)	Age, first abdominal pain time, initial pain site, RLQ abdomen shift, WBC, neutrophil count	The model was trained 10 times to assess reliability. Each time, an independent dataset was used for testing. Training took 135 s, while testing achieved diagnosis results in less than 1 s	Fuzzy-rule-based system: Accuracy rate (presence: 92%, high risk: 90%, reject: 87.5%, average: 89.9%)	US results: Sensitivity: 74% Specificity: 43%	1. The proposed evolutionary algorithm enhances the knowledge base in the fuzzy rule-based system 2. Optimizing algorithm boosts prediction accuracy 3. Successful classification of AA presence/absence in high-risk patients 4. Acceptable diagnostic performance achieved with limited input parameters 5. The model improves processing time and reduces treatment costs for diagnosis	1. Neural networks require a large set of features for accurate performance 2. The Alvarado Scoring system is less valid than other techniques 3 SVM need at least 10 factors for effective classification 4. Data overlap among the three classes 5. Time and cost constraints associated with collecting more extensive input factors for other models
Park et al. [12], 2015 (South Korea)	Pain location, migration of RLQ, tenderness of RLQ, rebound tenderness of RLQ, bowel sound, nausea, vomiting, temperature, WBC counts	MLNN trained using BP and LM algorithms with 2 hidden layers, 31 neurons each. Neuron count optimized by MSE. Layers had summation (linear) and activation (sigmoid) parts. RBF and PNN used Gaussian functions in 1 hidden layer. PNN output included Gaussian and competitive activation functions	MLNN model: Sensitivity: 99.5%, Specificity: 96.6%, PPV: 94.8%, NPV: 99.7%, Accuracy: 97.8%, AUC: 0.985 RBFNN model: Sensitivity: 100%, Specificity: 99.7%, PPV: 99.5%, NPV: 100%, Accuracy: 99.8%, AUC: 0.998 PNN model: Sensitivity: 100%, Specificity: 99.1%, PPV: 98.4%, NPV: 100%, Accuracy: 99.4%, AUC: 0.993	Alvarado score: Sensitivity: 23.2%, Specificity: 87.4%, PPV: 43.2%, NPV: 77.9%, Accuracy: 72.2%, AUC: 0.633	1. ANN structures showed strong diagnostic performance for appendicitis compared to Alvarado’s scoring 2. Potential for aiding junior surgeons in diagnosis 3. ANNs with objective input data may perform well in other regions	1. ANNs’ performance depends on training experience 2. No comparison with other ML algorithms 3. Imaging methods like CT and ultrasound are not incorporated 4. Generalizability due to large sample size not addressed 5. Real-world clinical application of ANNs not discussed
Safavi et al. [13], 2015 (Iran)	Age, sex, WBC, PCT, CRP, PMN	Employed trial-and-error method to optimize network structure for predicting AA presence. Used 2 hidden layers with 2–20 neurons in steps of 2. Created 2000 unique networks with varying structures. Best accuracy (88%) achieved with 4–8–4–1 structure (4 inputs, 8 neurons in first hidden layer, 4 in second, 1 in output)	MLP model: Sensitivity: 97.6%, Specificity: 41.2%, Accuracy: 88%, AUC: 0.875	WBC: Sensitivity: 85.5%, Specificity: 41.2%, Accuracy: 78%, AUC: 0.789 CRP: Sensitivity: 92.8%, Specificity: 11.8%, Accuracy: 79%, AUC: 0.655 PCT: Sensitivity: 55.42%, Specificity: 29.4%, Accuracy: 51%, AUC: 0.421 PMN: Sensitivity: 65.1%, Specificity: 58.8%, Accuracy: 64%, AUC: 0.663	1. The developed ANN model had higher diagnostic accuracy (88%) compared to other tests 2. Combining methods and using advanced techniques like ANN can enhance disease identification	1. The differences in results might be due to the specific population studied 2. While the neural network model offers high accuracy, its complexity might pose challenges in practical implementation
Lee et al. [14], 2013 (Southern Taiwan)	Age, gender, temperature, CRP, WBC, segment form, migration of abdominal pain, anorexia, nausea or vomiting, right lower quadrant pain, and rebound tenderness	Fivefold cross-validation with 6 repetitions for unbiased prediction performance assessment (average across 30 trials)	PEL model: Sensitivity: 57.3%, Specificity: 66.7%, AUC: 0.619	SVM model: Sensitivity: 100%, Specificity: 0.0%, AUC: 0.500 SMOTE model: Sensitivity: 70.1%, Specificity: 37.7%, AUC 0.539 MCC model: Sensitivity: 56.8%, Specificity: 58.9%, AUC: 0.579 CM model: Sensitivity: 56.1%, Specificity: 61.7%, AUC: 0.589 WCUS model: Sensitivity: 54.6%, Specificity: 58.2%, AUC: 0.564 Alvarado model: Sensitivity: 48.9%, Specificity: 61.0%, AUC: 0.580	1. Effectiveness in Imbalanced Learning: The PEL technique effectively handles imbalanced sample learning 2. Reduced Bias: PEL shows less bias toward either positive or negative classes compared to benchmark techniques 3. Superior Performance: PEL outperforms prevalent scoring systems and other classification techniques that use resampling	1. Incomplete Data 2. Limited Scope: Data are from one medical center and period 3. Narrow Focus: The study is specific to acute appendicitis and certain techniques 4. Limited Variables: Only quick laboratory test variables are considered 5. Negative Case Bias: Negative cases had surgery but different diagnoses
Yoldaş et al. [15], 2012 (Turkey)	Sex, intensity of pain, relocation of pain, pain in the right lower abdominal quadrant, vomiting, temperature, guarding, bowel sounds, rebound tenderness, WBC	Three-layered, multilayer perceptron ANN models, with BP circuit	ANN model: Sensitivity: 100%, Specificity: 97.2%, PPV: 88%, NPV: 100%, AUC: 0.95	NR	1. The ANNs technique is effective in diagnosing appendicitis 2. ANNs are particularly useful in rural hospitals where other diagnostic tools like US and CT scans are unavailable	1. Varying study populations affect results 2. Model variables are not cause-effect based 3. Single-center, limited data
Sun et al. [16]. 2012 (South Korea)	Features in univariate analysis: lymphocytes, urine glucose, total bilirubin, total amylase, chloride, red blood cells, neutrophils, eosinophils, white blood cells, complaints, basophils, glucose, monocytes, activated partial thromboplastin time, urine ketone, and direct bilirubin Features in multivariate analysis: neutrophils, complaints, total bilirubin, urine glucose, and lipase	The study employed DT models for the diagnosis of AA, utilizing statistical tests including univariate analysis and Wald forward LR with specific entry and removal criteria. To assess model performance, a tenfold cross-validation approach was implemented. The dataset was divided randomly into ten subsets, with nine used for training (90%) and one for testing (10%) in each iteration. This process was repeated ten times to ensure unbiased generalization error estimation	DT model: Accuracy: 78.9% DT model based on univariate analysis: Sensitivity: 82.4%, Specificity: 78.3%, PPV: 76.8%, NPV: 83.5%, AUC: 0.803, Accuracy: 80.2% DT model based on multivariate analysis: Sensitivity: 66%, Specificity: 80%, PPV: 74.3%, NPV: 72.9%, AUC: 0.73, Accuracy: 73.5%	NR	1. Development of a reliable hybrid DT model for early diagnosis of suspected AA 2. Potential application in supporting initial decisions by clinicians and increasing vigilance in suspected cases	1. Small sample size for acute and non-acute appendicitis 2. Potential variations in derived parameters and relationships 3. Lack of external validation or prospective studies
Hsieh et al. [17], 2011 (Taiwan)	Age, sex, migration of pain, anorexia, nausea/vomiting, RLQ tenderness, rebounding pain, diarrhea, progression of pain, right flank pain, body temperature, WBC, neutrophil (%), CRP, urine occult blood, hemoglobin	The study used a tenfold cross-validation for training and validation of each model. Default settings were first applied, and then adjusted for better performance. The RF model used 200 trees, SVM used nu-SVC type with polynomial kernel and probability estimates. ANN utilized a multilayer perception network with a BP algorithm, with specific settings for learning rate, momentum, and training time. The “nominaltobinaryfilter” parameter for ANN was set to false for optimization	RF model: AUC: 0.98 (0.017) Accuracy: 96%, Sensitivity: 94% Specificity: 100%, PPV: 100%, NPV: 87%, SVM model: AUC: 0.96 (0.027), Accuracy: 93%, Sensitivity: 91%, Specificity: 100%, PPV: 85%, NPV: 73%, ANN model: AUC: 0.91 (0.047), Accuracy: 91%, Sensitivity: 94%, Specificity: 85%, PPV: 94%, NPV: 85%, LR model: AUC: 0.87 (0.052), Accuracy: 82%, Sensitivity: 91%, Specificity: 62%, PPV: 85%, NPV: 73%	Alvarado: Sensitivity: 84%, Specificity: 69%, PPV: 87%, NPV: 64%), AUC: 0.77 (0.057), Accuracy: 80%, An Alvarado score of 6 was the best cutoff value for the prediction of AA (AC = 0.80, SN = 0.84, SP = 0.69)	1. RF outperforms other models in diagnosing AAP 2. The model offers an easy, fast, low-cost, and noninvasive diagnostic method 3. Weka’s open-source software allows for easy implementation 4. Web-based UI and compatibility with electronic medical records enable real-time, automated alerts for clinicians	1. Performance in other hospital settings is unproven 2. The complexity of the algorithm may limit its understanding and adoption by clinicians 3. Prospective external validation has not been performed
Ting et al. [18], 2010 (Taiwan)	Age, gender, migrating pain, anorexia, nausea, vomiting, RLQ tenderness, rebound pain, temperature, WBC, neutrophil count	A C5.0 DT algorithm developed by Quinlan with 3 decision levels and 6 leaf nodes was identified,	DT model: Sensitivity: 94.5%, Specificity: 80.5%	NR	1. Female patients with AA were older than males (*p* < 0.001) 2. No gender predominance among patients with normal appendices 3. Age was a risk factor for perforated appendicitis 4. Perforated cases had longer hospital stays and higher treatment costs 5. Alvarado’s scoring system did not differentiate well between acute and perforated appendicitis (*p* = 0.348)	1. No exploration of reasons behind older women’s higher risk 2. Lack of detail on cost factors or cost-reduction strategies 3. Absence of discussion on data collection biases or DT modeling limitations
Prabhudesai et al. [19], 2008 (UK)	Site of maximum pain, anorexia nausea, vomiting, site of tenderness, peritonism, temperature, WBC, neutrophil count, age, sex	The study employed various ML algorithms to model postoperative sepsis risk after appendectomy. This involved random weight assignment, training with retrospective data from 50 patients (25 with inflamed appendix), weight adjustment using a training algorithm, error correction through a BP algorithm, and weight fine-tuning to minimize MSE. Validation was conducted with data from an additional 20 patients. The ANN’s architecture was optimized, utilizing a single output node and empirically determined middle [2–15] and input [11] layer node numbers	ANN model: Sensitivity: 100%, Specificity: 97.2%, PPV: 96%, NPV: 100%	Alvarado (score ≥ 7): Sensitivity: 91.7%, Specificity: 83.3%, PPV: 78.6%, NPV: 93.8% Alvarado (score ≥ 6): Sensitivity: 95.8%, Specificity: 72.2%, PPV: 69.7%, NPV: 96.2% Clinical assessment: Sensitivity: 87.5%, Specificity: 80.5%, PPV: 75%, NPV: 90.6%	1. The ANN technique is effective in diagnosing appendicitis 2. It has the potential to reduce unnecessary explorations, negative appendectomy rates, and overall costs	1. The system’s efficiency is highly dependent on the accuracy of the knowledge base 2. Excessive variables may decrease the accuracy of the procedure 3. ANN improves diagnostic accuracy but cannot explain the reasoning behind its conclusions to the user
Sakai et al. [20], 2007 (Japan)	Gender, age, temperature, migration, tenderness at RLQ, rebound tenderness, muscular guarding, CRP, WBC	Feed-forward ANN models with three layers (input, hidden, output) and LR models were created using nine variables. Validation was done using the “.632 + bootstrap method” to evaluate accuracy	ANN model: Sensitivity: 76.7%, Specificity: 73.5%, PPV: 75%, NPV: 75.3%, AUC: 0.801 LR model: Sensitivity: 50%, Specificity: 92.8%, PPV: 87.8%, NPV: 64.2%, AUC: 0.774	Clinical diagnosis: Sensitivity: 100%, Specificity: 0%, PPV: 87.8%, NPV: 0%	1. The ANN model’s accuracy was better than the initial diagnosis based solely on clinical and laboratory findings 2. Reliance on imaging examinations like CT scans is still necessary for precise diagnosis	1. Single-institution study 2. Low proportion of key symptom (right lower quadrant tenderness)
Pesonen et al. [21], 1996 (Finland)	Demographics, initial pain characteristics, pain progression and factors, symptoms, physical examination, laboratory test	The algorithms underwent training using patient data and were tested using a separate patient test set to assess their performance and classification abilities	ART1 model: Sensitivity: 79%, Specificity: 78%, UI (usefulness index): 0.45, SOM mode: all parameters A (Sensitivity: 62%, Specificity: 82%, UI: 0.27) (all parameters B (Sensitivity: 55%, Specificity: 83%, UI: 0.21), LVQ model: (all parameters A (Sensitivity: 82%, Specificity: 87%, UI: 0.56) (all parameters B (Sensitivity: 87%, Specificity: 90%, UI: 0.68), BP model: (all parameters B (Sensitivity: 83%, Specificity: 92%, UI: 0.62)	NR	1. LVQ and BP algorithms are effective in diagnosing AA 2. Supervised learning outperforms unsupervised learning 3. Clinical signs are the best diagnostic parameters	1. Unsupervised learning lacks clinical sensitivity 2. Study limited to specific algorithms and parameters 3. Impact of using all clinical signs not explored
Forsstrom et al. [22], 1995 (Finland)	CRP, WBC, Phospholipase A2 (PLA2)	Used single-layer perceptron and CNN (BP network with 1 hidden layer) models. Each network and dataset were tested 3 times with random weights, learning factor 0.02, momentum 0.7, and 10,000 iterations	DiagaiD model: AUC: 0.6825, MSE: 0.0728 LR model: AUC: 0.677, SEM: 0.071 BP model (original data): 2 hidden nodes: (AUC: 0.6363 MSE: 0.0813, 3 hidden nodes: (AUC: 0.5537 MSE: 0.0819) 4 hidden nodes: (AUC: 0.6469 MSE: 0.0747) BP model (transformed data): 2 hidden nodes: (AUC: 0.6219 MSE: 0.0763), 3 hidden nodes: (AUC: 0.6069, MSE: 0.0756) 4 hidden nodes: (AUC: 0.6075, MSE: 0.0732)	NR	1. Neuro-fuzzy effective in clinical knowledge extraction 2. DiagaiD outperforms LR 3. Suitable for small datasets 4. Knowledge easily understood by clinicians	1. Risk of overlearning in large networks 2. Sharp cutoff values need adjustment 3. Requires 10 × cases per parameter for reliability 4. Further experiments are needed for parameter order

*DL* deep learning, *AUC* area under curve, *PPV* positive predictive value, *NPV* negative predictive value, *CNN* convolutional neural network, *AA* acute appendicitis, *RF* random forest, *NLP* natural language processing, *NA* no appendicitis, *NR* not reported, *Nor A* normal appendicitis, *DT* decision tree, *SVM* support vector machine, *ANN* artificial neural network, *FCM* fuzzy C-means clustering, *US* ultrasonography, *MLP* multilayer perceptron, *MLNN* multilayer neural network, *PNN* probabilistic neural network, *RBF* radial basis function, *SOM* self-organizing map, *BP* backpropagation, *LVQ* learning vector quantization, *LR* logistic regression, *PEL* pre-clustering based ensemble learning, *CA* complicated appendicitis, *UCA* uncomplicated appendicitis, *NB* Naïve Bayes, *SMOTE* synthetic minority oversampling technique, *MCC* Matthews correlation coefficient, *CM* cluster medoid, *WCUS* within cluster under-sampling, *FP* false positive, *TP* true positive, *WBC* white blood cell, *CRP* c-reactive protein, *PCT* procalcitonin, *PMN* polymorphic nuclear, *MSE* mean squared error, *PCC* percent correctly classified, *CHAID* chi-square automatic interaction detection, *RIF* right iliac fossa, *NIRIF* RIF pain with no inflammation, *IRIF* RIF pain with inflammation

#### Input Features

Each study employed a unique spectrum of input variables to train their models. A majority of the studies predominantly utilized the incorporation of demographic factors, clinical indicators, and laboratory measurements as the primary features for model training [21, 24, 26, 34, 35, 37, 39, 42, 44]. Radiological assessments, particularly CT images, were the chosen input modality in three studies [12, 20, 22]. Laboratory data served as the exclusive input for four studies [19, 23, 32, 36]. Additionally, three studies deployed a combination of clinical observations and laboratory data as their input features [38, 40, 41]. In one particular study, the input comprised a fusion of demographic and laboratory data [33]. Infrared thermographic evaluations of the abdomen were employed in a single study [31], while another study showcased a multivariable approach, incorporating demographic, clinical, laboratory, and ultra-sonographic data [30].

#### Performance Metrics

In each study, different measures were used to evaluate how well the models performed. The most frequently used metrics were sensitivity and specificity, appearing in 82% of the studies. Next in line was area under the curve (AUC) and accuracy, featured in 68% and 64% of studies, respectively. Positive predictive value (PPV) was used in 45% of the studies, and negative predictive value (NPV) in 41%. Less commonly, metrics like F1-score, usefulness index (UI), and mean squared error (MSE) were each used in just one study, making up 5% of all studies.

#### Performance

The comprehensive performance data for each model can be found in Table 2. The models’ performance underwent no types of validation in seven studies [34, 35, 37, 38, 40, 41, 43], underwent solely internal validation in 21 studies [3, 19–33, 39, 42, 44, 45], and was subject to both internal and external validation in only one study [12].

Numerous AI algorithms were employed in the diagnosis of AA, with ANN being the most commonly utilized in ten studies. Owing to the diversity in these algorithms, a direct performance comparison is not feasible. Regarding ANN and its variants, all studies that disclosed their accuracy rates reported figures exceeding 80%, peaking at 97.8% [38]. The AUCs ranged from 0.55 [32] to 0.985 [38].

In studies employing DT, the accuracy metrics span from 78.87 [36] to 84.4% [30] with AUC values ranging from 0.803 [36] to 0.93 [40]. As for studies that employed LR, the accuracy metrics were observed to vary from 82% [24] to 87.5% [30], and AUC values spanning from 0.677 [32] to 0.87 [24]. Regarding DL techniques, specifically CNN, accuracy rates were found to lie between 72.5 [20] and 97.5% [12]. The AUC values for these studies ranged from 0.724 [20] to 0.951 [22]. Supplementary parameters for these algorithms, as well as other less frequently utilized algorithms, are detailed in Table 2.

### Prognosis of Appendicitis

Eight articles in the review focused on the prognosis of AA. Six articles aimed to differentiate between complicated and uncomplicated cases [3, 19, 25, 27, 43, 45] and two studies focused on postoperative outcomes. One such study specifically analyzed the post-surgical outcomes of perforated appendicitis [29], while the other scrutinized the likelihood of sepsis following surgery, and its impact as a 30-day mortality risk factor [28]. Comprehensive information on the studies included in this domain is summarized in Tables 3 and 4.

**Table 3:** Characteristics of studies in artificial intelligence applications for appendicitis prognosis

Study, year	Objectives	Algorithms applied	Sample size (training: validating)	Age, mean ± SD (year)	Sex (male/female)
Akbulut et al. [2], 2023 (Turkey)	Develop an ML model for the classification of AA and predicting perforated and non-perforated AA	CatBoost model	1797 (80%/20%) (AA: 1465, NA: 332, AA non-perforated 1161, AA perforated: 304)	Male (median: 33; IQR: 23). female (median: 34; IQR: 26). AA (median: 33.1; IQR: 25), NA (median: 33; IQR: 24)	993/804
Tuong-Anh Phan-Mai et al. [23], 2023 (Vietnam)	Develop and validate ML models for detecting CA	SVM, DT, KNN, LR, ANN, and GB	1950 (483 CA, 1467 UCA)	Totally: 37:3 ± 15:9, CA: 40:6 ± 17:3, UCA: 36:2 ± 15:2	Total: 678/826, CA: 233/250, UCA: 652/815
Li et al. [24], 2023 (China)	Develop a scoring system using clinical and imaging features to differentiate CA from UCA in pregnant individuals	LR, DT	342 patients, 141 (41.23%) were diagnosed with CA, and 201 (58.77%) were diagnosed with UCA	Totally: 27.78 ± 3.36 CA: 27.87 ± 4.62 UCA: 27.72 ± 4.7	All patients were female
Lin et al. [25], 2023 (Taiwan)	Assess the ability of ANN models, to distinguish between UCA and CA	ANN (MLP)	411 AA patients (288 (253 UCA, 35 CA): 123 (109 UCA, 14 CA)	Totally: 43.9 ± 17 Training set: 43.6 ± 16.5 Testing set: 44.8 ± 18.1	Totally: 206/205 Training set: 144/144 Testing set: 62/61
Eickhof et al. [26], 2022 (Germany)	Create and validate an ML model for predicting postoperative outcomes of perforated appendicitis	RF classification is based on stratified under-sampling, i.e., an ensemble of DT	163 (64 patients underwent laparoscopic surgery, 99 patients got an open procedure)	38.1 ± 26.3	92/71
Xia et al. [27], 2022 (China)	Develop an accurate, rapid, noninvasive, and cost-effective diagnostic rule to differentiate between CA and UCA	OBLGOA-SVM, GOA-SVM, GS-SVM, RF, ELM, KELM, BPNN	298 (150 UCA, 148 CA)	UCA: 42.23 ± 15.54, CA: 46.57 ± 19.73	NR
Kang et al. [28], 2021 (China)	Develop ML models to predict the pathological types of AA preoperatively	LR	136 (acute SA = 8, acute PA = 104, acute GPA = 24). The sample size was divided 70/30 for training and testing SA/PA (112): training: 78, testing: 34. PA/GPA (128), training: 89, testing: 39	SA: 39.12 ± 20.00, PA: 42.02 ± 17.31, GPA: 40.54 ± 15.31	SA: 5/3, PA: 56/48, GPA: 12/11
Corinne Bunn et al. [29], 2021 (USA)	Apply different ML algorithms to predict the risk of postoperative sepsis after appendectomy, assess their effectiveness, and identify related risk factors	Multivariable LR, SVM, RFDT, and extreme gradient boosting (XGB)	223,214 records for appendectomy (221,073 had no postoperative sepsis, 2141 had postoperative sepsis)	Postoperative sepsis: 48.09 ± 18.41 No postoperative sepsis: 39.8 ± 16.3	Postoperative sepsis: 58.7%/41.3 No postoperative sepsis: 50.8%/49.2%

*ML* machine learning, *AA* acute appendicitis, *IQR* interquartile range, *NA* no appendicitis, *SVM* support vector machine, *DT* decision tree, *KNN* k-nearest neighbor, *LR* logistic regression, *ANN* artificial neural network, *GB* gradient boosting, *CA* complicated appendicitis, *UCA* uncomplicated appendicitis, *SA* simple appendicitis, *PA* purulent appendicitis, *GPA* gangrenous or perforated appendicitis, *RF* random forest, *GOA* Grasshopper Optimization Algorithm, *NR* not reported, *BNC* Bayesian network classifiers, *RFDT* random forest decision tree, *OBLGOA* opposition based learning grasshopper optimization algorithm, *GS* grid search, *ELM* extreme learning machine, *KELM* kernel extreme learning machine, *BPNN* backpropagation neural network

**Table 4:** Details of artificial intelligence methods applied and outcomes in studies for appendicitis prognosis

Study, year	Input features	Training/validation strategy	Performance	Comparative algorithms and scoring metrics	Key findings	Limitation
Akbulut et al. [2], 2023 (Turkey)	Neutrophil, WLR, NLR, CRP, WNR, PNR, PDW, and MCV	The persistence method was repeated 50 times with different seeds for model robustness. CatBoost model predicted AA and perforated AA, with optimized hyperparameters using grid search with tenfold cross-validation and 5 replicates	CatBoost model performance for classification: Sensitivity 84.2%, Specificity 93.2%, AUC 0.947, Accuracy 88.2%, F1-score 88.7% CatBoost model: Accuracy 0.92, F1-score 91.1%, Sensitivity 94.1%, Specificity 90.5%, and AUC 0.969	NR	1. First study to combine ML and XAI for AA and perforated AA estimation 2. Identified biochemical blood parameters that can predict AA and perforated AA	1. The study is retrospective and lacks comprehensive clinical data 2. Radiological data are missing for approximately 11% of the patient sample 3. Conducted at a single institution
Phan-Mai et al. [23], 2023 (Vietnam)	Demographic characteristics, blood tests, and ultrasound. Blood tests consisted of total WBC, granulocyte count, lymphocyte count, and CRP	Imbalanced data was addressed using SMOTE. Optimal parameters were selected using k-fold validation. The data of 1,950 patients were split randomly into 70% for training and 30% for testing	GB model (imbalanced unadjusted data): Accuracy: 81%, AUC: 0.753 GB model (imbalanced adjusted data): Accuracy: 82%, AUC: 0.890 KNN model (imbalanced unadjusted data): Accuracy: 77.6%, AUC: 0.672, KNN model (imbalanced adjusted data): Accuracy: 74.1%, AUC: 0.831 DT model (imbalanced unadjusted data): Accuracy: 70.3%, AUC: 0.601 DT model (imbalanced adjusted data): Accuracy: 73.8%, AUC: 0.738 ANN model (imbalanced unadjusted data): Accuracy: 80.5%, AUC: 0.734 ANN model (imbalanced adjusted data): Accuracy: 74.2%, AUC: 0.810 LR model (imbalanced unadjusted data): Accuracy: 80.3%, AUC: 0.714 LR model (imbalanced adjusted data): Accuracy: 72.9%, AUC: 0.789 SVM model (imbalanced unadjusted data): Accuracy: 75.2%, AUC: 0.711 SVM model (imbalanced adjusted data): Accuracy: 65.5%, AUC: 0.730	NR	1. High validity of ML models in classifying CA 2. GB model most valid 3. Models useful as screening tools	1. Small sample size 2. Single-hospital data 3. Low rate of complicated cases 4. Insufficient qualitative data 5. Not for definitive diagnosis
Li et al.[24], 2023 (China)	age, stage of pregnancy; symptom duration time, vital signs, physical examination findings; laboratory test results; and image findings (US)	NR	LR based score (Cutoff = 16) Sensitivity: 64%, Specificity: 84%, Accuracy: 75%, PPV: 73%, NPV: 77%, AUC: 0.80 (95% CI = 0.75–0.84) DT model: AUC: 0.78	NR	1. Higher premature birth and abortion rates in pregnant patients with CA 2. Treatment delay increases these rates 3. Models using LR and DT effectively distinguish CA from UCA 4. Models combine clinical and laboratory tests 5. Appendix diameter had an AUC of 0.68 in 116 cases	1. Single-center study 2. No external validation 3. Limited patient number 4. Appendix diameter not included
Lin et al. [25], 2023 (Taiwan)	CRP level, NLR, CT findings (fat-stranding sign, appendicolith, and ascites)	The data preprocessing involved standardizing independent variables AA patients to a scale of 0 to 1. Patients were then randomly divided into training and testing datasets at a 70:30 ratio. A single hidden layer with three neurons was chosen using a predefined value to avoid overfitting, as it was sufficient for the dataset	ANN model (MLP): AUC: 0.950, Sensitivity: 85.7%, Specificity: 91.7%, LR + : 10.36, LR-: 0.16	NR	1. A three-layer MLP with three hidden neurons performed well 2. Practical application would require an integrated system for immediate predictions after a CT scan	1. Single-center study 2. Broad definition of complicated appendicitis 3. Potential variation in definitions across studies
Eickhoff et al. [26], 2022 (Germany)	Age, gender, height, weight, and BMI, clinical-anamnestic data such as the ASA score, comorbidities, and perioperative data (time interval from admission to appendectomy, operative time, hemoglobin, CRP, WBC, platelets, INR, open surgery, laparoscopic surgery, conversion, extended surgical procedures during appendectomy, drains) as predictor variables	The dataset was split into 10 equal parts. 90% was used for training and 10% for validation. This process was repeated for all sections of the data, rotating the test sample. This was done 50 times for stable performance assessment	RF model: Need for ICU (Accuracy: 77.2%, Sensitivity: 77.9%, Specificity: 76.9% Longer stay > 24 h in ICU (Accuracy: 87.5%, Sensitivity: 88.4%, Specificity: 87.4%) Complications measured by Clavien-Dindo > 3 in new cases (Accuracy: 68.2%, Sensitivity: 61.6%, Specificity: 69.5%) Re-operation after initial appendectomy (Accuracy: 74.2%, Sensitivity: 47.5%, Specificity: 77.2% occurrence of surgical site infection (Accuracy: 66.4%, Sensitivity: 66.2%, Specificity: 66.4%) Need for oral antibiotic therapy after discharge (Accuracy: 78.8%, Sensitivity: 76.4%, Specificity: 79.1%) More than 7 days of hospital stay (Accuracy: 76.2%, Sensitivity: 74.3%, Specificity: 77.9%) More than 15 days of hospital stay (Accuracy: 83.6%, Sensitivity: 60%, Specificity: 85.1%)	NR	1. Developed ML model for post-op outcomes in perforated appendicitis 2. The model predicts the need for intensive care 3. Suggests early transfer to higher-level care facilities	1. Single-center, retrospective study 2. Small sample size
Xia et al. [27], 2022 (China)	Gender, age, temperature, heart rate, WBC, lymphocytes, neutrophils, monocytes, eosinophils, hemoglobin, erythrocytes, platelets, urea nitrogen, blood sugar, creatinine, bilirubin, CRP	Used tenfold cross-validation for overall classification evaluation, and fivefold cross-validation for parameter optimization. Assessed using 12 benchmark functions	OBLGOA-SVM model: Accuracy: 83.6%, MCC: 67.3%, Sensitivity: 81.7%, Specificity: 85.3%	GOA-SVM model: Accuracy: 81%, MCC: 64%, Sensitivity: 78% Specificity: 84% GS-SVM model: Accuracy: 79%, MCC: 59%, Sensitivity: 72%, Specificity: 86% RF model: Accuracy: 82%, MCC: 65%, Sensitivity: 82%, Specificity: 82% ELM model: Accuracy: 77%, MCC: 55%, Sensitivity: 72%, Specificity: 81% KELM model: Accuracy: 78%, MCC: 57%, Sensitivity: 71%, Specificity: 84% BPNN model: Accuracy: 76%, MCC: 52%, Sensitivity: 75%, Specificity: 76%	1. Proposed OBLGOA-SVM framework for CA vs. UCA 2. Improved GOA for SVM parameters 3. Method outperformed rivals in evaluations 4. CRP, heart rate, temp, and neutrophils predict CA	1. No radiological findings (ultrasound, CT scans) 2. Insufficient cases from a single center 3. Uncontrolled, retrospective study
Kang et al. [28], 2021 (China)	Age, gender, clinical signs and symptoms score, abdominal pain score, vomiting score, abdominal pain time, abdominal pain type, abdominal tenderness pain range, and the highest temperature. laboratory records: blood routine, coagulation function, blood biochemistry, WBC, NE, CD3 + T, CD4 + T, CD8 + T, CD19 + T, CD16 + 56, NK, total T cell counts, helper T cell counts, inhibitors T, B cell counts, NK cell counts, CD4 + /CD8 + ratio, CRP, PCT, and blood NLR ratio	LR models were created separately for SA/PA and PA/GPA groups using selected features from the training dataset. Clinical features were added to establish combined LR models. Models were then validated using testing sets	LR model: Acute SA vs. PA (based on T cell subsets alone): training set (AUC: 0.904, Accuracy: 87.5%, Sensitivity: 75%, Specificity: 100%), testing set (AUC: 0.910, Accuracy: 87.5%, Sensitivity: 75%, Specificity: 100%), Acute SA versus acute PA (based on T cell subsets and clinical signs and symptoms): training set (AUC: 0.921, Accuracy: 91%, Sensitivity: 81.9%, Specificity: 100%) testing set (AUC: 0.926, Accuracy: 90.6%, Sensitivity: 81.2%, Specificity: 100%), Acute PA vs. acute GPA (based on T cell subsets alone): training set (AUC: 0.834, Accuracy: 82.6%, Sensitivity: 81.9%, Specificity: 83.3%) testing set (AUC: 0.821, Accuracy: 80.6%, Sensitivity: 90.3%, Specificity: 71%), Acute PA vs. acute GPA (based on T cell subsets and clinical signs and symptoms) training set: (AUC: 0.867, Accuracy: 80.6%, Sensitivity: 73.6%, Specificity: 87.5%), testing set (AUC: 0.854, Accuracy: 77.4%, Sensitivity: 90.3%, Specificity: 64.5%)	NR	1. Established a quick diagnosis model using peripheral blood biomarkers for AA pathology	1. Limited cases 2. Single-center data source 3. The study could not fully prove biomarkers’ predictive value due to sample size and false positives
Corinne Bunn et al. [29], 2021 (USA)	Demographic, comorbid conditions, preoperative laboratory results, days, and procedure-related information	The dataset is split into 80% training and 20% hidden testing. Missing data imputed using multivariable imputation for complete analysis	Postoperative sepsis prediction LR model: AUC: 0.69, Sensitivity: 62%, Specificity: 65% SVM model: AUC: 0.51 RFDT model: AUC: 0.69, Sensitivity: 67%, Specificity: 60% XGB model: AUC: 0.70, Sensitivity: 64%, Specificity: 66% Ensemble model (LR, RFDT, and XGB): AUC: 0.70, Sensitivity: 64%, Specificity: 60% Postoperative sepsis prediction as a risk factor for 30-day mortality: LR model: AUC: 0.92, Sensitivity: 82%, Specificity: 87% SVM model: AUC: 0.5 RFDT model: AUC: 0.96, Sensitivity: 93%, Specificity: 84% XGB model: AUC: 0.93, Sensitivity: 89%, Specificity: 85% Ensemble model (LR, RFDT, and XGB): AUC: 0.95, Sensitivity: 89%, Specificity: 89%	NR	1. ML methods predict postoperative sepsis after appendectomy with moderate accuracy 2. Risk factors for postoperative sepsis: recent CHF exacerbation, acute renal failure, preoperative transfusion	1. High false positive rates in clinical implementation 2. The study focuses on non-septic cases, isolating early-stage disease 3. Missing intraoperative findings data 4. ML is used on a national database, not EHR data 5. ML does not outperform LR due to dataset quality

*ML* machine learning, *AA* acute appendicitis, *IQR* interquartile range, *NA* no appendicitis, *SVM* support vector machine, *DT* decision tree, *KNN* k-nearest neighbor, *LR* logistic regression, *ANN* artificial neural network, *GB* gradient boosting, neural network, *RFDT* random forest decision tree, *RBF* radial basis function, *SOM* self-organizing map, *BP* backpropagation, *LVQ* learning vector quantization, *PEL* pre-clustering based ensemble learning, *CA* complicated appendicitis, *UCA* uncomplicated appendicitis, *SA* simple appendicitis, *PA* purulent appendicitis, *GPA* gangrenous or perforated appendicitis, *NB* Naïve Bayes, *SMOTE* synthetic minority oversampling technique, *MCC* Matthews correlation coefficient, *WBC* white blood cell, *CRP* c-reactive protein, *PCT* procalcitonin, *PMN* polymorphic nuclear, *MSE* mean squared error, *SA* simple appendicitis, *PA* purulent appendicitis, *GPA* gangrenous or perforated appendicitis, *XAI* explainable artificial intelligence, *ROC* receiver operating characteristics, *US* ultrasonography, *WLR* white cell count lymphocyte ratio, *WNR* within normal range, *NLR* neutrophil to lymphocyte ratio, *ASA* American Society of Anesthesiologists Classification, *BMI* body mass index, *OBLGOA* opposition based learning grasshopper optimization algorithm, *GS* grid search, *ELM* extreme learning machine, *KELM* kernel extreme learning machine, *BPNN* backpropagation neural network, *LR+* positive likelihood ratio, *LR−* negative likelihood ratio

#### Predicting the Type of Appendicitis

Six studies examined the classification of appendicitis into complicated (CA) or uncomplicated (UCA) categories, all using laboratory results as a key input. Two of these investigations employed a blend of demographic, clinical, and laboratory data as their input data [3, 45]. Another study utilized a mixture of ultrasound observations alongside demographic, clinical, and laboratory data [43]. Additionally, one study focused on the integration of ultrasonography with demographic and laboratory findings [25], and yet another utilized CT data in conjunction with laboratory outcomes as their input variables [27].

Various algorithms were employed across the research for the categorization of appendicitis types. ANN [25, 27, 45] and LR [3, 25, 43] were the predominant methods, each featured in three studies. Following these, SVM [25, 45], DT [25], and ensemble techniques (GB [25, 43] and CatBoost [19]) were each implemented in two studies. Methods such as KNN [25], RF [45], ELM [45], and KELM [45] were each utilized in a single study.

In a majority of the studies, sensitivity [3, 19, 27, 43, 45], specificity [3, 19, 27, 43, 45], and AUC [3, 19, 25, 27, 43] emerged as the most commonly employed performance measures. Accuracy [3, 19, 25, 43] was highlighted in four research works. Singular studies made use of additional metrics such as F1-score [19], PPV [43], NPV [43], positive and negative likelihood ratios [27], as well as the Matthew Correlation Coefficient (MCC) [45].

Among the scrutinized studies, the study by Lin et al. [27] stood out for achieving an AUC of 0.950 by integrating both laboratory results and CT findings to forecast the type of AA. On a similar note, Akbulut et al. [19] employed CatBoost algorithms to forecast the types of appendicitis and achieved an AUC of 0.947 along with an 88.2% accuracy rate, solely based on laboratory findings as input variables. In a comparative analysis, Li et al. [43] demonstrated that LR outperformed DT in terms of AUC. A study by Phan-Mai et al. [25] highlighted that the GB algorithm yielded the most robust performance, attaining an accuracy rate of 82%. Similarly, Xia et al. [45] verified that the Opposition-Based Learning Grasshopper Optimization Algorithm (OBLGOA), a specialized form of SVM, achieved an accuracy of 83.5%. Kang et al. [3] employed LR to distinguish among different forms of appendicitis such as simple appendicitis (SA), perforated appendicitis (PA), and gangrenous perforated appendicitis (GPA), either relying exclusively on T cell data or coupling it with clinical information. In the context of differentiating SA from PA, the model that integrated both T cell metrics and clinical findings yielded a superior accuracy of 90.6%, compared to 87.5% when relying solely on T cell data. Conversely, when the objective was to differentiate PA from GPA, the model that exclusively utilized T cell data outstripped the composite-input model, with respective accuracy rates of 80.6% and 77.4%.

#### Predicting Postoperative Outcomes

Two studies focused on outcomes following surgery [28, 29]. In the investigation led by Eickhoff et al. [29], the RF algorithm was used to assess post-surgical outcomes. Various types of data were employed for predictive analysis, including patient demographics, clinical history, and perioperative data such as the interval between hospital admission and appendectomy, surgery duration, laboratory test results, type of surgery such as open or laparoscopic methods, conversions, additional procedures conducted during the appendectomy, and the use of surgical drains. The model’s performance varied across distinct clinical outcomes. It achieved an accuracy of 77.2% for the requirement of ICU admission, 87.5% for an extended ICU stay exceeding 24 h, and 68.2% for complications identified by the Clavien-Dindo score greater than 3 in newly diagnosed cases. Additionally, the likelihood of reoperation following the initial appendectomy was forecasted with a 74.2% accuracy rate, while surgical site infection rates were anticipated at 66.4% accuracy. The model also predicted the necessity for oral antibiotic treatment post-discharge with 78.8% accuracy, and hospital stays lasting more than 7 and 15 days were forecasted with accuracies of 76.2% and 83.6%, respectively. For model evaluation, accuracy, sensitivity, and specificity were used as the key performance indicators. Notably, the model demonstrated strong predictive capabilities for extended ICU stays greater than 24 h and for hospitalizations exceeding 15 days, achieving accuracies above 80% for these particular outcomes.

In research carried out by Corinne Bunn et al. [28], they assessed the likelihood of developing sepsis following an appendectomy and its contribution to mortality within 30 days. Various AI techniques were employed, with performance metrics compared across models. The feature set incorporated demographic data, pre-existing medical conditions, pre-surgical laboratory results, days, and details about the surgical procedure itself. Models utilized in their analysis included SVM, LR, XGB, and Random Forest Decision Trees (RFDT). Additionally, they created an ensemble model by amalgamating RFDT, LR, and XGB. The ensemble model and XGB demonstrated superior efficacy in forecasting post-appendectomy sepsis risk. While both models matched in terms of accuracy and sensitivity, the ensemble model lagged in specificity. RFDT and LR performed similarly but were surpassed by the aforementioned models, with SVM trailing in last place.

When evaluating sepsis as a contributing factor to 30-day mortality, RFDT took the lead in model performance. Following RFDT, the models ranked in descending order of effectiveness were: the ensemble model, XGB, LR, and finally SVM.

## Discussion

Appendicitis represents a surgical emergency demanding swift and precise diagnosis to avert potentially life-threatening complications such as peritonitis and sepsis [46]. AI algorithms have emerged as a transformative tool, significantly enhancing diagnostic accuracy and prognostic capabilities, thus potentially revolutionizing AA management [47]. In this systematic review, focused on AA in adult patients, we observe that the majority of research endeavors (72%) have been dedicated to diagnostic applications. This emphasis underscores the immediate clinical need for accurate AA diagnosis.

While diagnostic applications have dominated the landscape, a discernible surge in prognostic studies has emerged over the last 3 years. This trend reflects an evolving recognition of AI’s potential to diagnose and forecast patient outcomes, providing valuable insights for treatment strategies.

A diverse array of AI algorithms has been applied in both the diagnosis and prognosis of AA. These include ANN, SVM, DT, LR, RF, DL (CNN), and various other algorithms. Notably, traditional diagnostic approaches, such as the Alvarado scoring system, ultrasound findings, Eskelinen score, laboratory assessments, and clinical evaluation, have been included in some studies for comparative purposes. Encouragingly, AI consistently outperformed these traditional scoring systems, highlighting the superiority of ML algorithms in medical diagnosis and prognosis.

However, it is important to acknowledge the challenges in directly comparing these AI models. The considerable heterogeneity among studies, encompassing factors like single-center designs, small sample sizes, retrospective methodologies, variations in input features, diverse algorithms, and differing performance metrics, precluded a meta-analysis. This complexity underscores the need for caution when drawing quantitative comparisons between the various models.

Within the realm of studies employing multiple AI algorithms, ANN and its subtypes emerge as the top performers for diagnosing AA, demonstrating their robustness and versatility in this clinical context.

Moreover, individual studies have contributed unique insights. For instance, Rajpurkar et al. [20] demonstrated that CNN achieved superior performance when pretrained on video sequences, showcasing the potential benefits of data augmentation techniques. Sun et al. [36] highlighted that DT yielded more favorable results when combined with univariate analysis rather than multivariate analysis, underlining the importance of optimizing algorithm combinations.

In the prognostic domain, CatBoost demonstrated a stronger performance in prognosis compared to diagnosis, as evident in one study [19].

Timely and accurate diagnosis of appendicitis during pregnancy is crucial to minimize perinatal and maternal morbidity and mortality, yet is often delayed due to prevalent gastrointestinal symptoms and challenges in interpreting clinical and laboratory findings. Anatomical and physiological alterations, such as the displacement of the appendix by the enlarging uterus and pregnancy-induced leukocytosis, exacerbate these diagnostic difficulties, resulting in accurate preoperative diagnosis in merely 1/2 to 3/4 of cases [48]. In light of these diagnostic challenges, the selection of appropriate predictive algorithms becomes paramount. For instance, when predicting the type of appendicitis during pregnancy, LR outperforms DT, underscoring the importance of algorithm selection in enhancing diagnostic accuracy in specific clinical contexts [43].

A specific focus by Gudelis et al. [40] on ANN and DT revealed that both algorithms performed similarly for differential diagnoses of right iliac fossa pain, but ANN was significantly more effective for the diagnosis of AA.

Examining postoperative consequences in the context of perforated appendicitis [29], the RF algorithm consistently achieved an accuracy exceeding 70% across various clinical endpoints. These included admission to the ICU, ICU stays exceeding 24 h, the need for post-appendectomy reoperation, prescription of oral antibiotics following discharge, and extended hospital stays. These findings underscore the potential of AI to contribute to postoperative decision-making and patient care.

In the prediction of post-appendectomy sepsis risk [28], both the ensemble model (XGB + RFDT + LR) and XGB demonstrated high effectiveness, with similar accuracy and sensitivity. However, the ensemble model exhibited lower specificity. When assessing sepsis as a contributing factor to 30-day mortality, RFDT emerged as the top-performing model, offering valuable insights into postoperative patient management.

Our systematic review offers distinct advantages. We have incorporated both diagnostic and prognostic studies, providing a holistic view of AI’s role in adult AA management. This comprehensive approach enhances our understanding of AI’s clinical potential. By employing the PROBAST tool, we have critically evaluated the risk of bias, offering readers a transparent assessment of study reliability. Furthermore, our review furnishes detailed insights into the performance of various AI algorithms, aiding healthcare professionals and researchers in selecting suitable models for specific clinical applications. Additionally, by identifying limitations and research gaps, our review serves as a guiding compass for future investigations, emphasizing the importance of robust study designs and enhanced methodological rigor.

The findings of this systematic review hold significant practical implications for the field of appendicitis management. With AI algorithms consistently outperforming traditional diagnostic methods, clinicians may consider integrating these advanced tools into their decision-making processes. This could lead to more accurate and timely diagnoses, potentially reducing the risk of misdiagnoses and unnecessary surgeries. Moreover, the insights gained from prognostic studies could aid in tailoring treatment plans to individual patient needs, optimizing postoperative care, and improving outcomes. As for future research, these practical implications emphasize the importance of further investigating the implementation of AI in clinical settings, considering factors such as user-friendliness, accessibility, and cost-effectiveness to ensure real-world utility.

Despite its strengths, our systematic review does have limitations. The inherent heterogeneity among the included studies, characterized by factors such as single-center designs, limited sample sizes, retrospective methodologies, variations in input features, diverse AI algorithms, varying performance metrics, and outcome measures, hindered our ability to conduct a meta-analysis and draw direct quantitative comparisons between models. Furthermore, a majority of the reviewed studies exhibited a high risk of bias, particularly in terms of selection bias and the absence of internal validation, potentially impacting the generalizability of the evaluated AI models. Additionally, the dynamic nature of AI in healthcare suggests that new research may emerge, potentially influencing or altering our current findings over time.

While this systematic review provides a comprehensive overview of the current landscape, it also highlights several areas where future research can make valuable contributions. Firstly, studies that directly compare different AI algorithms in controlled clinical settings could provide insights into which algorithms perform best under specific conditions, facilitating algorithm selection for clinicians. Additionally, investigating the integration of AI systems into electronic health records and clinical workflows is crucial to ensure seamless adoption in healthcare settings. Furthermore, exploring the long-term impact of AI-driven decision support systems on patient outcomes and healthcare costs is an avenue for research. Finally, as AI technologies continue to evolve, ongoing research should focus on adapting these tools to emerging diagnostic and prognostic challenges in appendicitis management, ultimately enhancing patient care and safety.

## Conclusion

In conclusion, the application of AI in the context of appendicitis holds immense promise. It has already demonstrated its potential to significantly enhance diagnostic accuracy and prognostic capabilities, marking a transformative shift in how we approach this critical medical condition. The robust performance of various AI algorithms, outperforming traditional diagnostic methods, underscores their relevance in clinical practice.

### Supplementary Information

**Additional file 1**. PRISMA 2020 Checklist.

**Additional file 2**. Search Strategy for Four Databases.

**Additional file 3**. **Figure S1**. Study Selection.

**Additional file 4**. **Figure S2**. Frequency of AI research in appendicitis across various years.

**Additional file 5**. Assessment of the Included Studies Using PROBAST.

## Data Availability

All data are included in the main manuscript and supplemental files.
