# Supplementary material for: Artificial Intelligence and Acute Appendicitis: A Systematic Review of Diagnostic and Prognostic Models
Source: World J Emerg Surg. 2023 Dec 19;18:59. doi: 10.1186/s13017-023-00527-2 (PMC10729387; doi:10.1186/s13017-023-00527-2)
Supplement: Supplementary file 2 — Additional file 2. Search Strategy for Four Databases. [file 13017_2023_527_MOESM2_ESM.docx]

**PubMed**

("Artificial intelligence" OR "AI" OR "Machine learning" OR "Deep learning" OR "Neural networks" OR "Natural language processing" OR "Computer vision" OR "Pattern recognition" OR "Expert systems" OR "Cognitive computing") AND ("Appendicitis" OR "Acute appendicitis" OR "Appendicular inflammation" OR "Inflamed appendix" OR "Appendiceal infection" OR "Appendiceal abscess" OR "Appendiceal perforation" OR "Perforated appendix" OR "Appendiceal obstruction" OR "Appendiceal rupture")

**Web of Science**

TS=("Artificial intelligence" OR AI OR "Machine learning" OR "Deep learning" OR "Neural networks" OR "Natural language processing" OR "Computer vision" OR "Pattern recognition" OR "Expert systems" OR "Cognitive computing") AND TS=("Appendicitis" OR "Acute appendicitis" OR "Appendicular inflammation" OR "Inflamed appendix" OR "Appendiceal infection" OR "Appendiceal abscess" OR "Appendiceal perforation" OR "Perforated appendix" OR "Appendiceal obstruction" OR "Appendiceal rupture")

**Scopus**

TITLE-ABS-KEY("Artificial intelligence" OR "AI" OR "Machine learning" OR "Deep learning" OR "Neural networks" OR "Natural language processing" OR "Computer vision" OR "Pattern recognition" OR "Expert systems" OR "Cognitive computing") AND TITLE-ABS-KEY("Appendicitis" OR "Acute appendicitis" OR "Appendicular inflammation" OR "Inflamed appendix" OR "Appendiceal infection" OR "Appendiceal abscess" OR "Appendiceal perforation" OR "Perforated appendix" OR "Appendiceal obstruction" OR "Appendiceal rupture ")

**Embase**

('artificial intelligence'/exp OR 'artificial intelligence' OR ai OR 'machine learning'/exp OR 'machine learning' OR 'deep learning'/exp OR 'deep learning' OR 'neural networks'/exp OR 'neural networks' OR 'natural language processing'/exp OR 'natural language processing' OR 'computer vision'/exp OR 'computer vision' OR 'pattern recognition'/exp OR 'pattern recognition' OR 'expert systems'/exp OR 'expert systems' OR 'cognitive computing'/exp OR 'cognitive computing') AND ('appendicitis'/exp OR appendicitis OR 'acute appendicitis'/exp OR 'acute appendicitis' OR 'appendicular inflammation'/exp OR 'appendicular inflammation' OR 'inflamed appendix' OR 'appendiceal infection' OR 'appendiceal abscess'/exp OR 'appendiceal abscess' OR 'appendiceal perforation'/exp OR 'appendiceal perforation' OR 'perforated appendix' OR 'appendiceal obstruction' OR 'appendiceal rupture'/exp OR 'appendiceal rupture')
