# Supplementary figures and images for: Artificial Intelligence and Acute Appendicitis: A Systematic Review of Diagnostic and Prognostic Models
Source: World J Emerg Surg. 2023 Dec 19;18:59. doi: 10.1186/s13017-023-00527-2 (PMC10729387; doi:10.1186/s13017-023-00527-2)

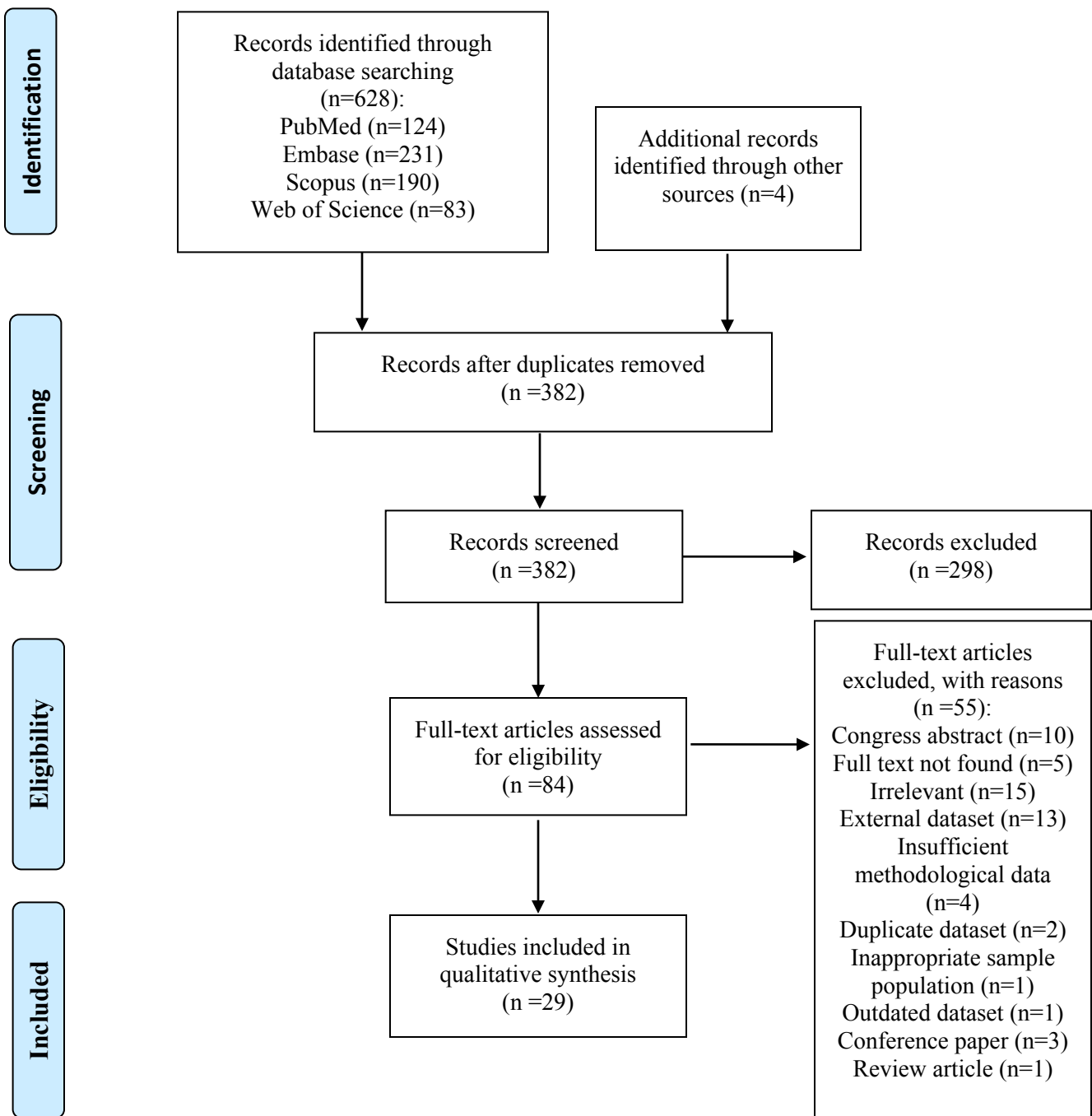

Supplement: Supplementary file 3 — Additional file 3. Figure S1. Study Selection. [file 13017_2023_527_MOESM3_ESM.pdf]

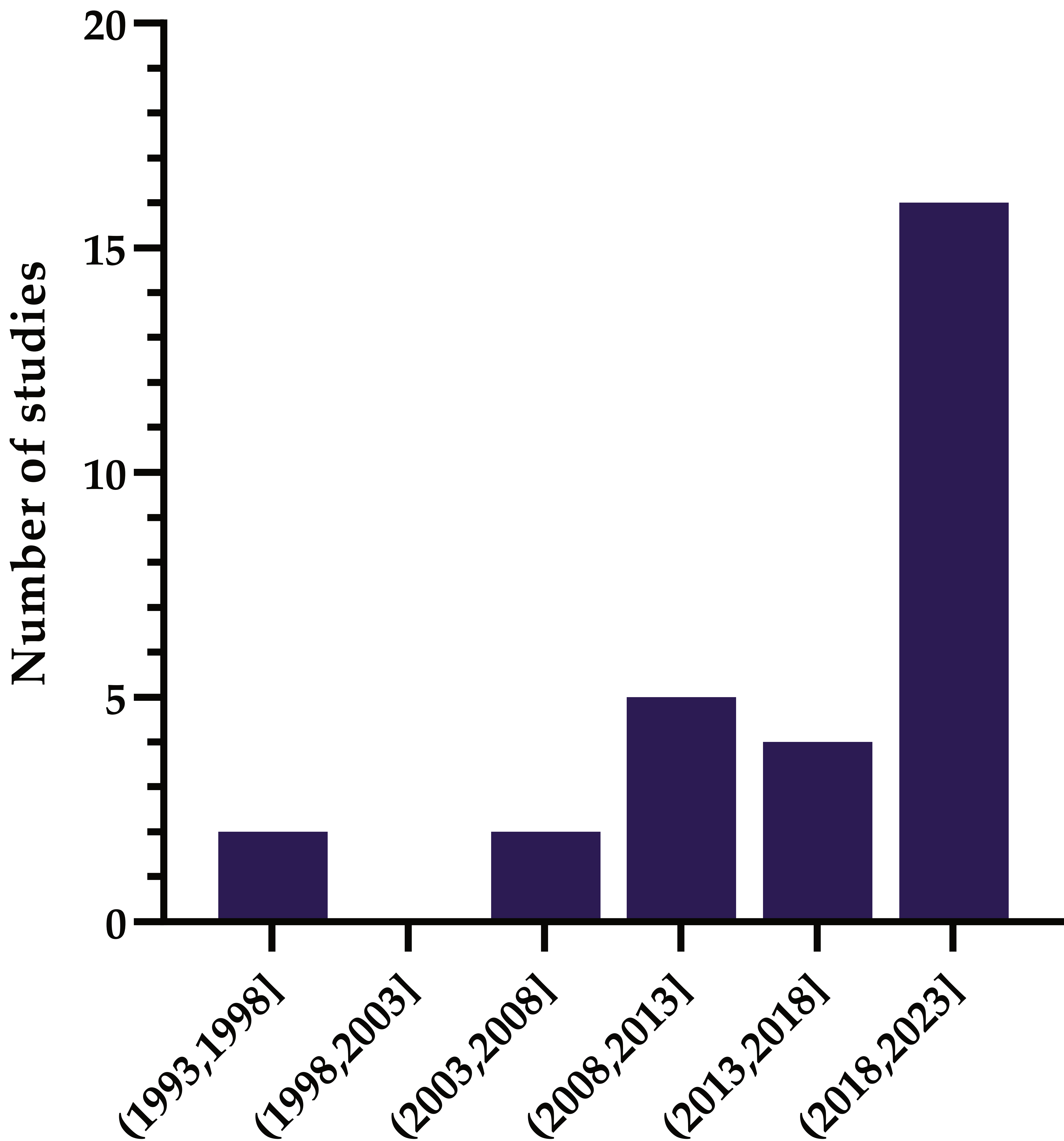

Supplement: Supplementary file 4 — Additional file 4. Figure S2. Frequency of AI research in appendicitis across various years. [file 13017_2023_527_MOESM4_ESM.pdf]
