# Supplementary material for: Artificial Intelligence and Acute Appendicitis: A Systematic Review of Diagnostic and Prognostic Models
Source: World J Emerg Surg. 2023 Dec 19;18:59. doi: 10.1186/s13017-023-00527-2 (PMC10729387; doi:10.1186/s13017-023-00527-2)
Supplement: Supplementary file 5 — Additional file 5. Assessment of the Included Studies Using PROBAST. [file 13017_2023_527_MOESM5_ESM.docx]

| **Study Info.** | | | | **PROBAST Step-2** | **DOMAIN-1: Participants Risk of Bias** | **DOMAIN-1: Participants Applicability** | **DOMAIN-2: Predictors Risk of Bias** | **DOMAIN-2: Predictors Applicability** | **DOMAIN-3: Outcome Risk of Bias** | **DOMAIN-3: Outcome Applicability** | **DOMAIN-4: Analysis** | **Step-4: Overall Assessment** | |
| --- | --- | --- | --- | --- | --- | --- | --- | --- | --- | --- | --- | --- | --- |
| **#** | **Title** | **First author** | **Date** | **Classify the type of prediction model evaluation** | **RISK** | **CONCERN** | **RISK** | **CONCERN** | **RISK** | **CONCERN** | **RISK** | **RISK** | **CONCERN** |
| **1** | Convolutional-neural-network- based diagnosis of appendicitis via CT scans in patients with acute abdominal pain presenting in the emergency department | Park JJ | 2020 | Dev. & Validation (int. & ext. val.) | 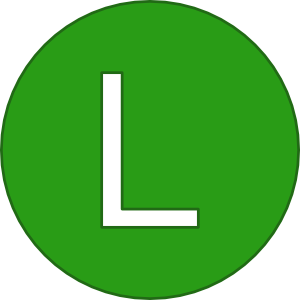 | 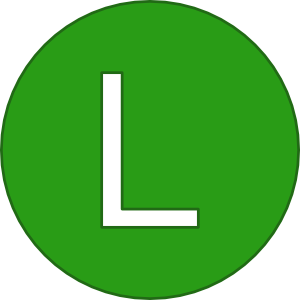 | 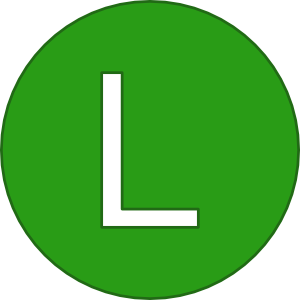 | 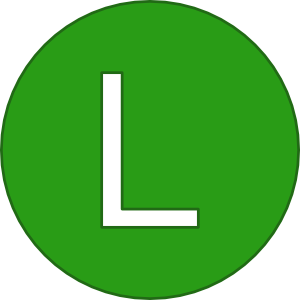 | 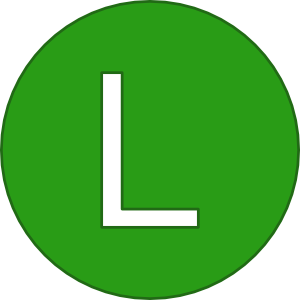 | 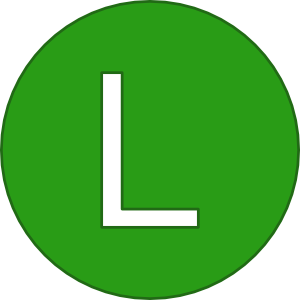 | 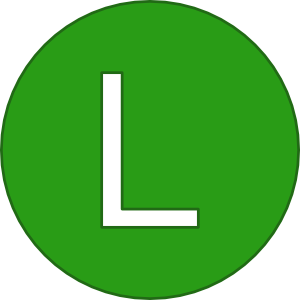 | 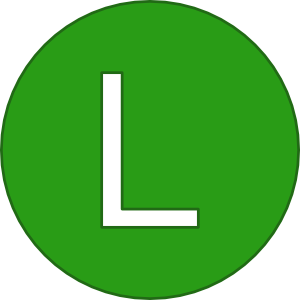 | **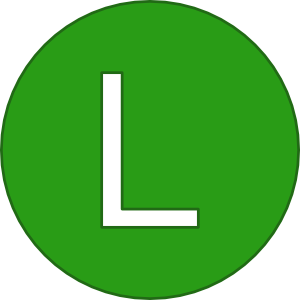** |
| **2** | A preclustering-based ensemble learning technique for acute appendicitis diagnoses | Lee YH | 2013 | Development (int. val.) | 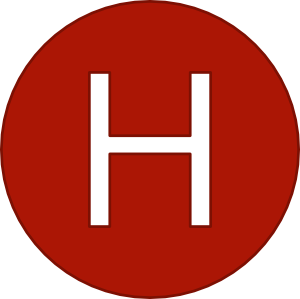 | 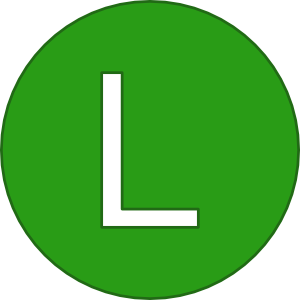 | 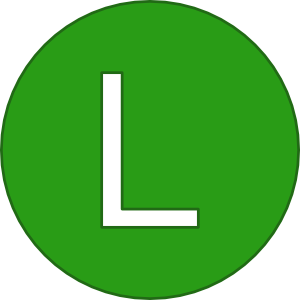 | 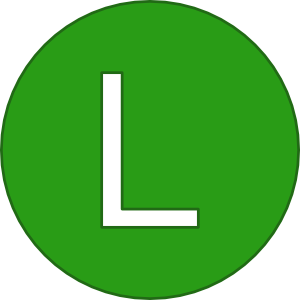 | 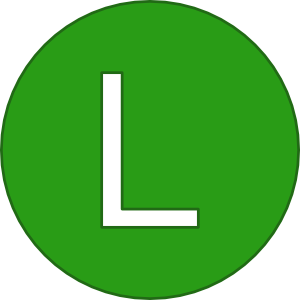 | 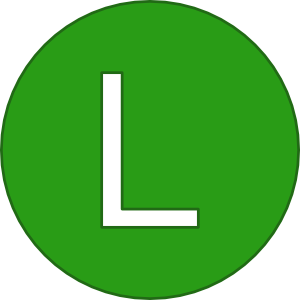 | 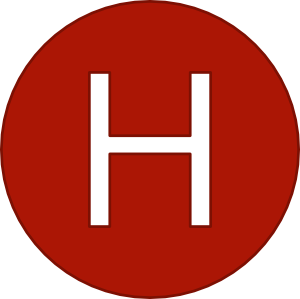 | 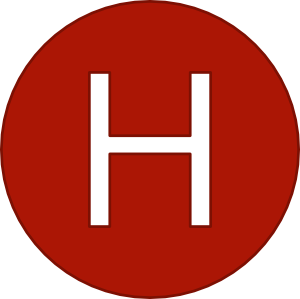 | **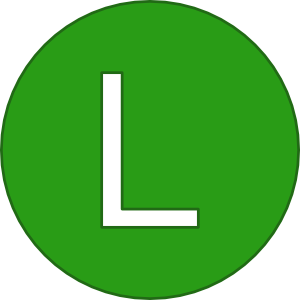** |
| **3** | AppendiXNet: Deep Learning for Diagnosis of Appendicitis from A Small Dataset of CT Exams Using Video Pretraining | Rajpurkar P | 2020 | Development (int. val.) | 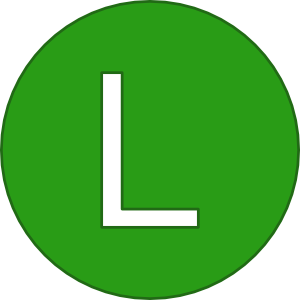 | 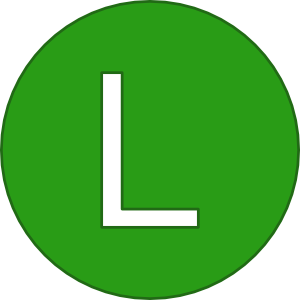 | 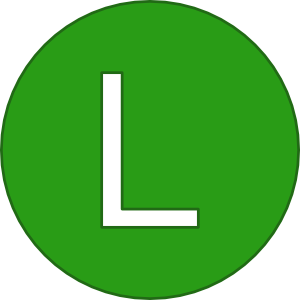 | 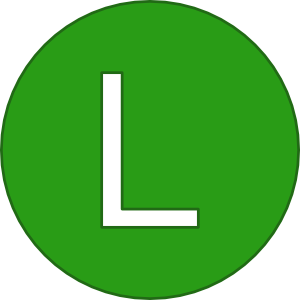 | 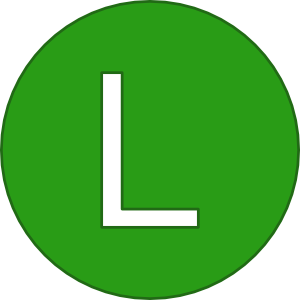 | 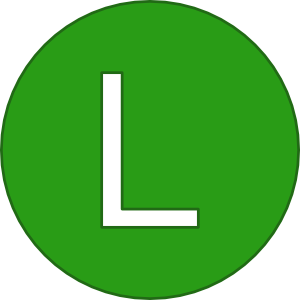 | 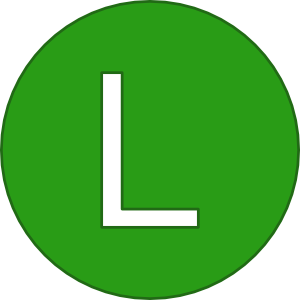 | 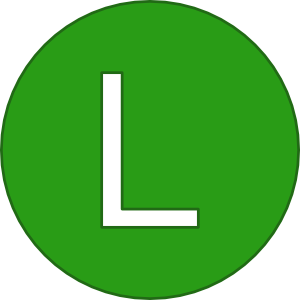 | **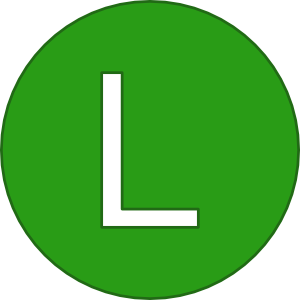** |
| **4** | Application of machine learning to the prediction of postoperative sepsis after appendectomy | Bunn C | 2020 | Development (int. val.) | 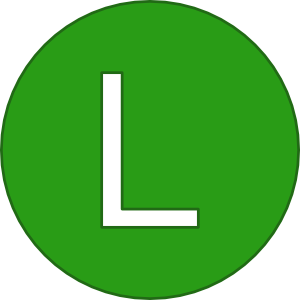 | 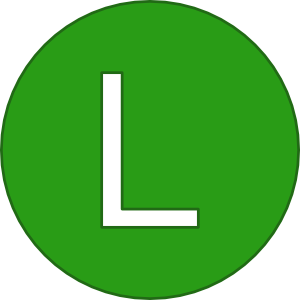 | 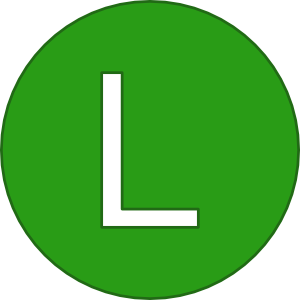 | 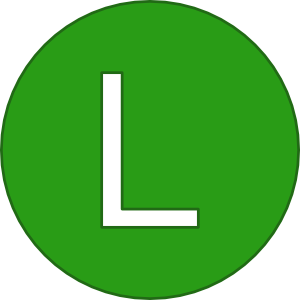 | 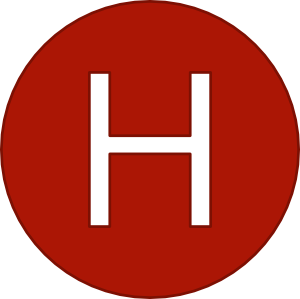 | 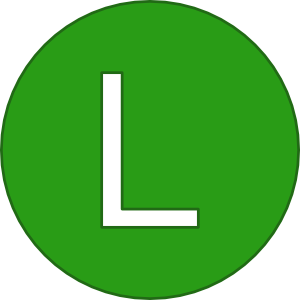 | 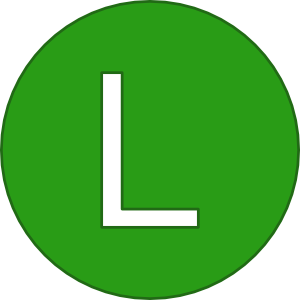 | 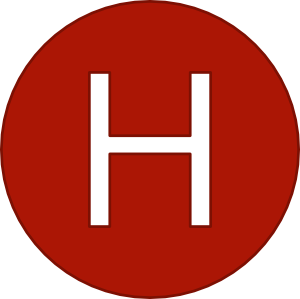 | **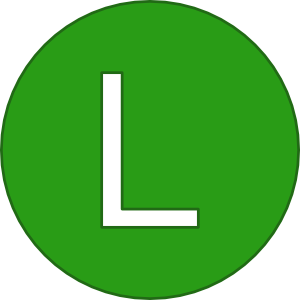** |
| **5** | Artificial Neural Networks: Useful Aid in Diagnosing Acute Appendicitis | Prabhudesai SG | 2008 | Development (int. val.) | 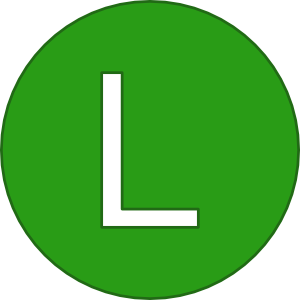 | 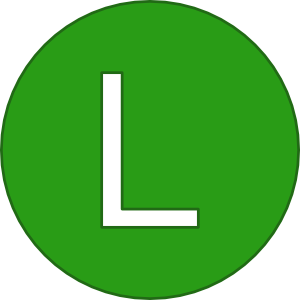 | 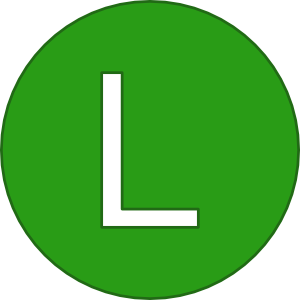 | 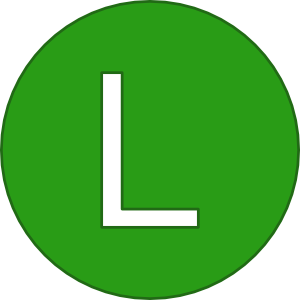 | 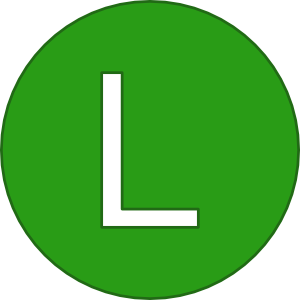 | 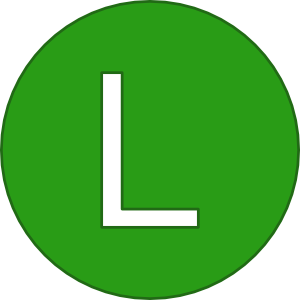 | 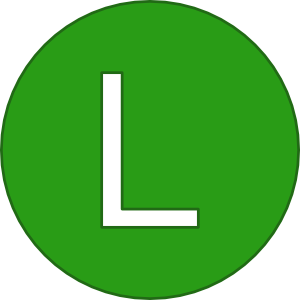 | 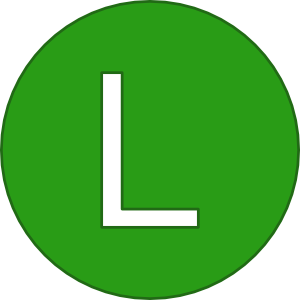 | **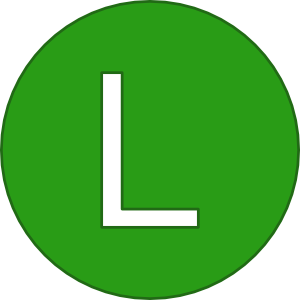** |
| **6** | Comparing the Accuracy of Neural Network Models and Conventional Tests in Diagnosis of Suspected Acute Appendicitis | Afshari Safavi A | 2015 | Development (int. val.) | 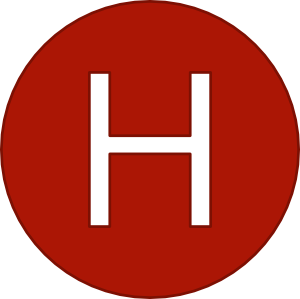 | 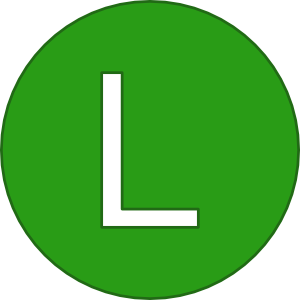 | 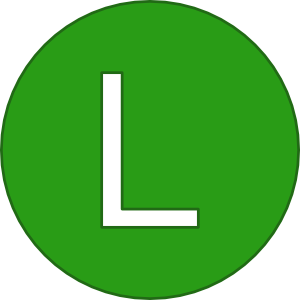 | 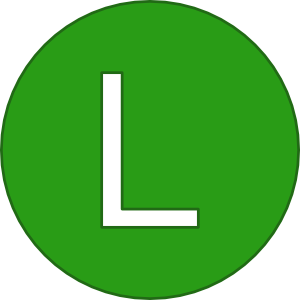 | 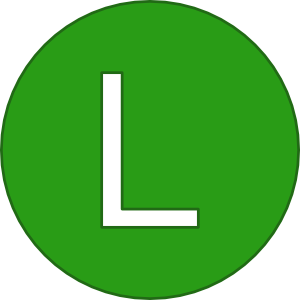 | 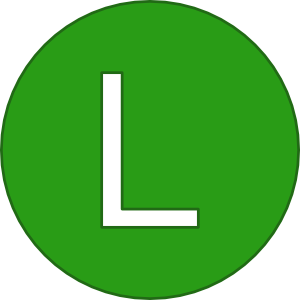 | 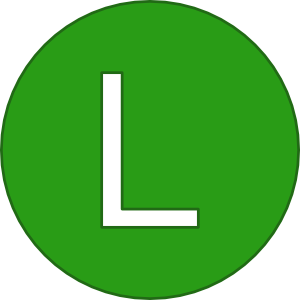 | 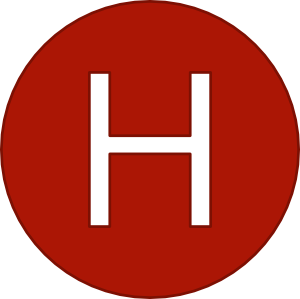 | **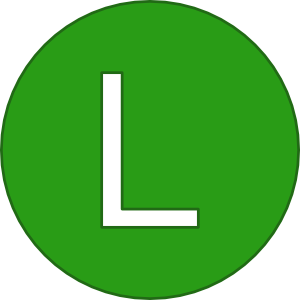** |
| **7** | Comparison between single and serial computed tomography images in classification of acute appendicitis, acute right-sided diverticulitis, and normal appendix using EfficientNet | Park SH | 2023 | Development (int. val.) | 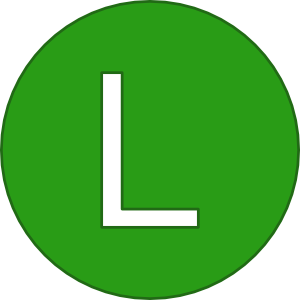 | 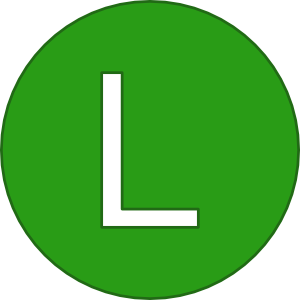 | 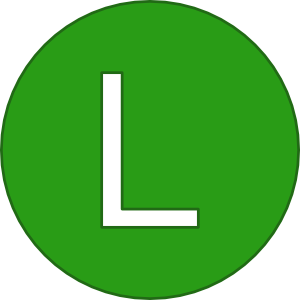 | 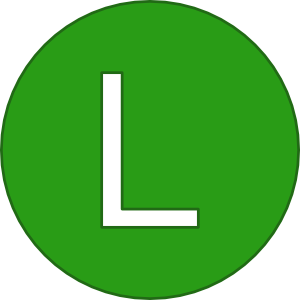 | 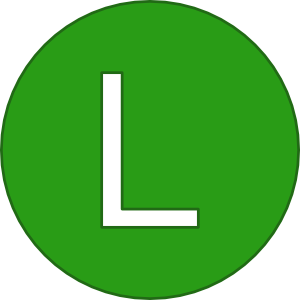 | 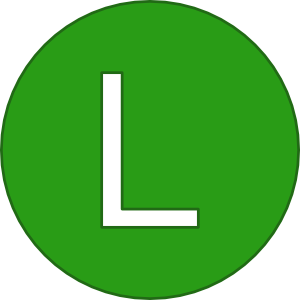 | 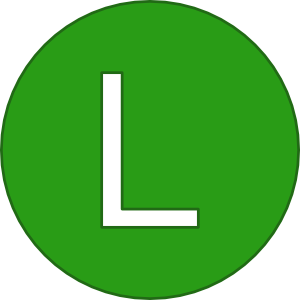 | 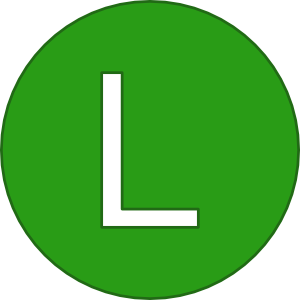 | **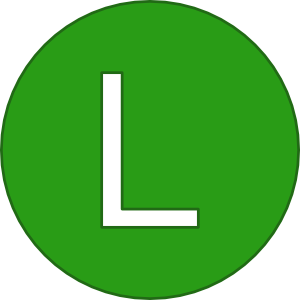** |
| **8** | Comparison of the Levels of Accuracy of an Artificial Neural Network Model and a Logistic Regression Model for the Diagnosis of Acute Appendicitis | Sakai S | 2007 | Development (int. val.) | 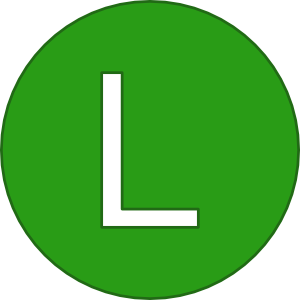 | 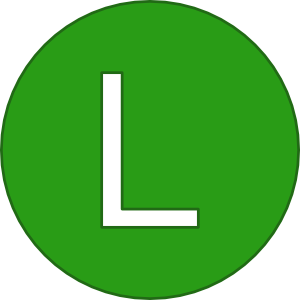 | 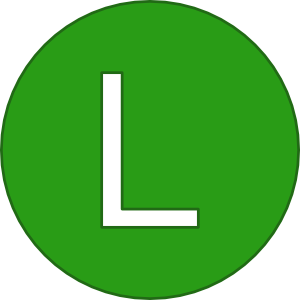 | 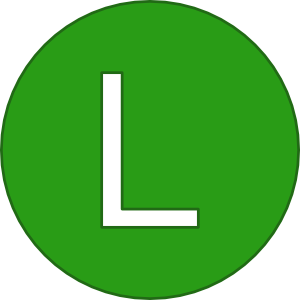 | 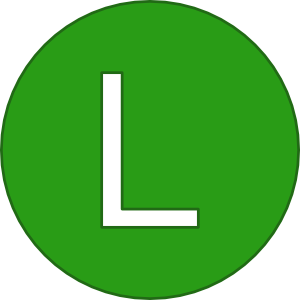 | 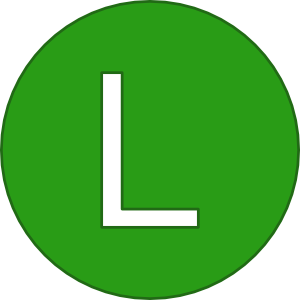 | 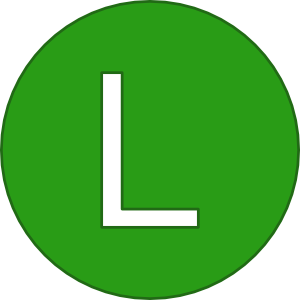 | 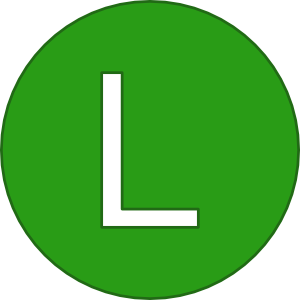 | **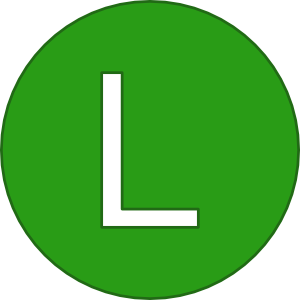** |
| **9** | Discovery of Urinary Proteomic Signature for Differential Diagnosis of Acute Appendicitis | Zhao Y | 2020 | Development (int. val.) | 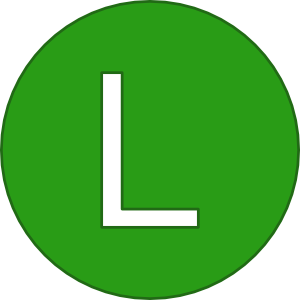 | 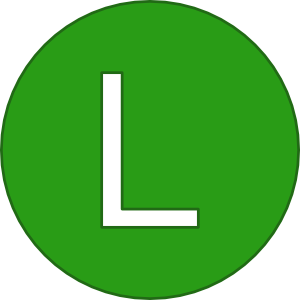 | 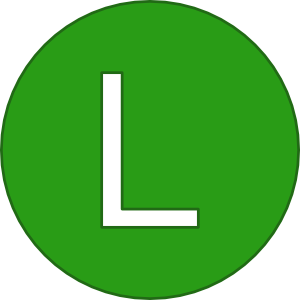 | 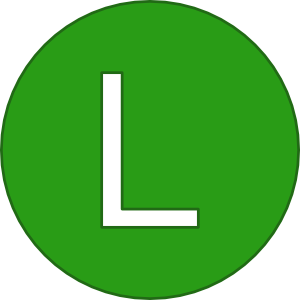 | 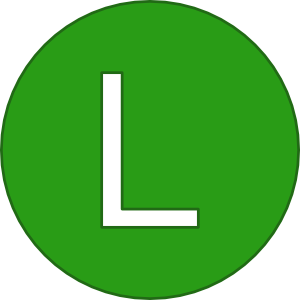 | 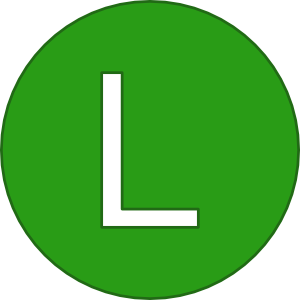 | 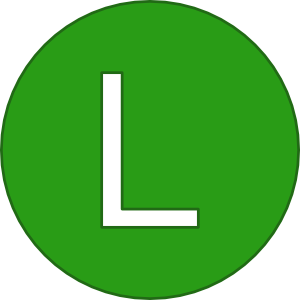 | 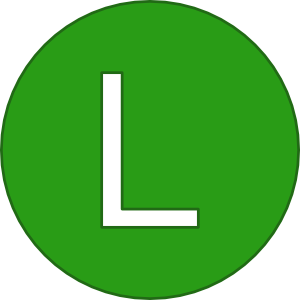 | **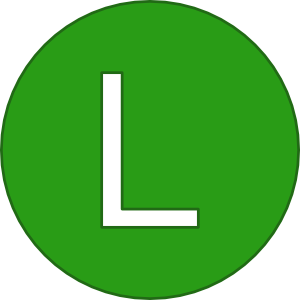** |
| **10** | Infrared thermography of abdominal wall in acute appendicitis: Proof of concept study | Ramirez-GarciaLunaa JL | 2020 | Development (int. val.) | 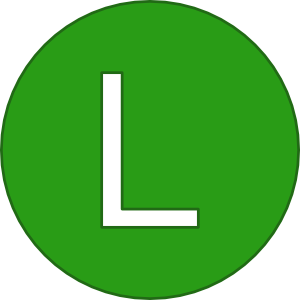 | 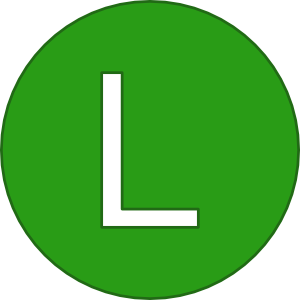 | 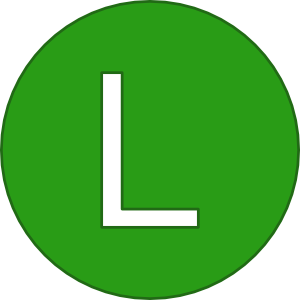 | 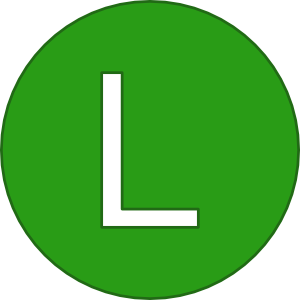 | 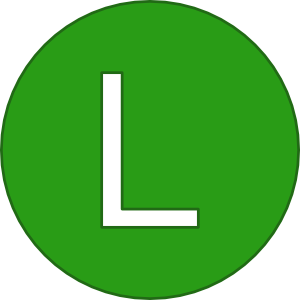 | 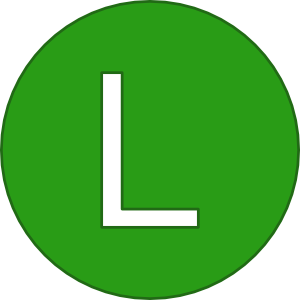 | 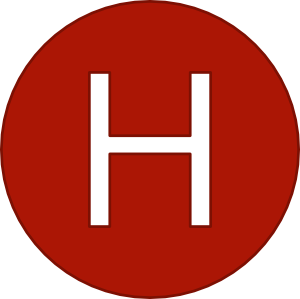 | 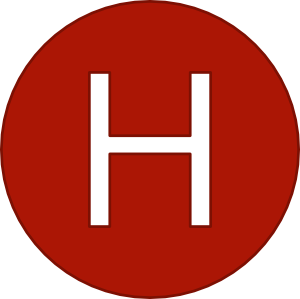 | **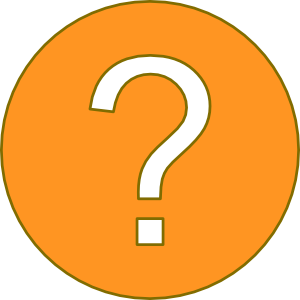** |
| **11** | Machine learning prediction model for postoperative outcome after perforated appendicitis | Eickhoff RM | 2022 | Development (int. val.) | 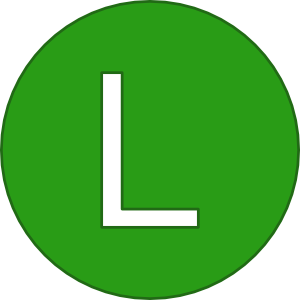 | 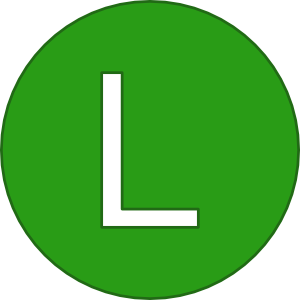 | 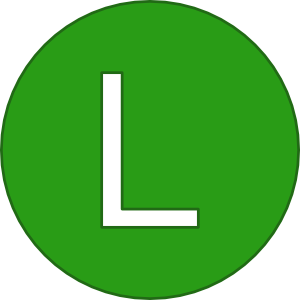 | 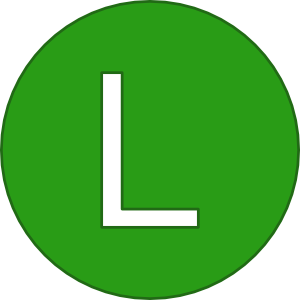 | 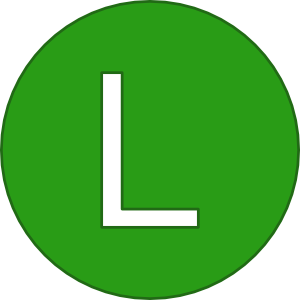 | 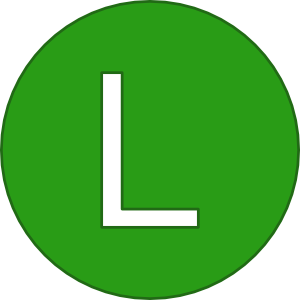 | 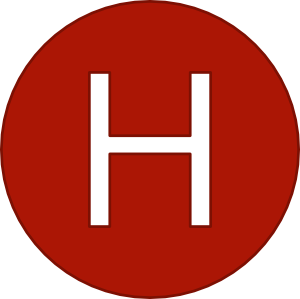 | 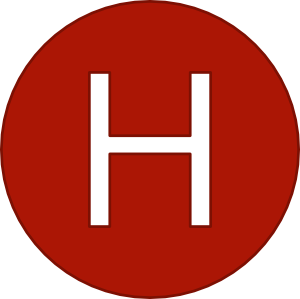 | **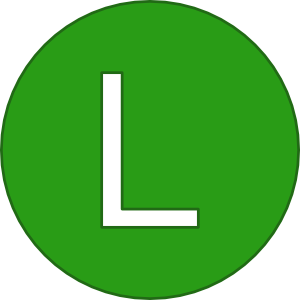** |
| **12** | Novel solutions for an old disease: Diagnosis of acute appendicitis with random forest, support vector machines, and artificial neural networks | Hsieh CH | 2010 | Development (int. val.) | 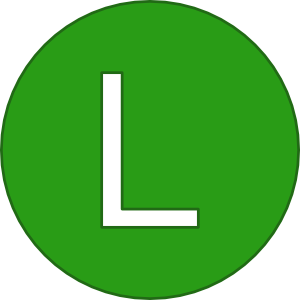 |  |  |  |  |  |  |  |  |
| **13** | Performance Optimization of Support Vector Machine with Oppositional Grasshopper Optimization for Acute Appendicitis Diagnosis | Xia J | 2022 | Development (int. val.) |  |  |  |  |  |  |  |  |  |
| **14** | Predicting Risk of Acute Appendicitis: A Comparison of Artificial Neural Network and Logistic Regression Models | Shahmoradi L | 2018 | Development (int. val.) |  |  |  |  |  |  |  |  |  |
| **15** | Prediction of Perforated and Nonperforated Acute Appendicitis Using Machine Learning-Based Explainable Artificial Intelligence | Akbulut S | 2023 | Development (int. val.) |  |  |  |  |  |  |  |  |  |
| **16** | Preoperatively predicting the pathological types of acute appendicitis using machine learning based on peripheral blood biomarkers and clinical features: a retrospective study | Kang CB | 2021 | Development (int. val.) |  |  |  |  |  |  |  |  |  |
| **17** | Using data preprocessing and single layer perceptron to analyze laboratory data | Forsström JJ | 1995 | Development (int. val.) |  |  |  |  |  |  |  |  |  |
| **18** | Validity of Machine Learning in Detecting Complicated Appendicitis in a Resource-Limited Setting: Findings from Vietnam | Phan-Mai TA | 2023 | Development (int. val.) |  |  |  |  |  |  |  |  |  |
| **19** | A Computer Based Model in Comparison with Sonography Imaging to Diagnosis of Acute Appendicitis in Iran | Jamshidnezhad A | 2017 | Development (int. val.) |  |  |  |  |  |  |  |  |  |
| **20** | Artifcial Intelligence Compared to Alvarado Scoring System Alone or Combined with Ultrasound Criteria in the Diagnosis of Acute Appendicitis | Ghareeb WM | 2021 | Development (int. val.) |  |  |  |  |  |  |  |  |  |
| **21** | Application of Artificial Neural Network Models to Differentiate Between Complicated and Uncomplicated Acute Appendicitis | Lin HA | 2023 | Development (int. val.) |  |  |  |  |  |  |  |  |  |
| **22** | A hybrid decision support model to discover informative knowledge in diagnosing acute appendicitis | Son CS | 2012 | Development (int. val.) |  |  |  |  |  |  |  |  |  |
| **23** | Acute appendicitis diagnosis using artificial neural networks | Park SY | 2015 | No Internal Validation | N/A | N/A | N/A | N/A | N/A | N/A | N/A | N/A | N/A |
| **24** | Artificial neural networks in the diagnosis of acute appendicitis | Yoldaş Ö | 2012 | No Internal Validation | N/A | N/A | N/A | N/A | N/A | N/A | N/A | N/A | N/A |
| **25** | Comparison of different neural network algorithms in the diagnosis of acute appendicitis | Pesonen E | 1996 | No Internal Validation | N/A | N/A | N/A | N/A | N/A | N/A | N/A | N/A | N/A |
| **26** | Establishment of predictive models for acute complicated appendicitis during pregnancy—A retrospective case–control study | Li P | 2022 | No Internal Validation | N/A | N/A | N/A | N/A | N/A | N/A | N/A | N/A | N/A |
| **27** | Evaluation of the diagnostic performance of a decision tree model in suspected acute appendicitis with equivocal preoperative computed tomography findings compared with Alvarado, Eskelinen, and adult appendicitis scores A STARD compliant article | Kang HJ | 2019 | No Internal Validation | N/A | N/A | N/A | N/A | N/A | N/A | N/A | N/A | N/A |
| **28** | Diagnosis of Pain in the Right Iliac Fossa. A New Diagnostic Score Based on Decision-Tree and Artificial Neural Network Methods§ | Gudelis M | 2019 | No Internal Validation | N/A | N/A | N/A | N/A | N/A | N/A | N/A | N/A | N/A |
| **29** | Decision Model for Acute Appendicitis Treatment with Decision Tree Technology—A Modification of the Alvarado Scoring System | Ting HW | 2010 | No Internal Validation | N/A | N/A | N/A | N/A | N/A | N/A | N/A | N/A | N/A |
